# Methods for identification of spike patterns in massively parallel spike trains

**DOI:** 10.1007/s00422-018-0755-0

**Published:** 2018-04-12

**Authors:** Pietro Quaglio, Vahid Rostami, Emiliano Torre, Sonja Grün

**Affiliations:** 1 Institute of Neuroscience and Medicine (INM-6) and Institute for Advanced Simulation (IAS-6), JARA Institute Brain Structure-Function Relationships (INM-10), Jülich Research Centre, Jülich, Germany; 2 Computational Systems Neuroscience, Institute for Zoology, Faculty of Mathematics and Natural Sciences, University of Cologne, Cologne, Germany; 3 Chair of Risk, Safety and Uncertainty Quantification, ETH Zürich, Zurich, Switzerland; 4 Risk Center, ETH Zürich, Zurich, Switzerland; 5 Theoretical Systems Neurobiology, RWTH Aachen University, Aachen, Germany

**Keywords:** Spike synchrony, Spatio-temporal spike patterns, Correlated point processes, Monte Carlo methods, Data mining

## Abstract

Temporally, precise correlations between simultaneously recorded neurons have been interpreted as signatures of cell assemblies, i.e., groups of neurons that form processing units. Evidence for this hypothesis was found on the level of pairwise correlations in simultaneous recordings of few neurons. Increasing the number of simultaneously recorded neurons increases the chances to detect cell assembly activity due to the larger sample size. Recent technological advances have enabled the recording of 100 or more neurons in parallel. However, these massively parallel spike train data require novel statistical tools to be analyzed for correlations, because they raise considerable combinatorial and multiple testing issues. Recently, various of such methods have started to develop. First approaches were based on population or pairwise measures of synchronization, and later led to methods for the detection of various types of higher-order synchronization and of spatio-temporal patterns. The latest techniques combine data mining with analysis of statistical significance. Here, we give a comparative overview of these methods, of their assumptions and of the types of correlations they can detect.

**Electronic supplementary material:**

The online version of this article (10.1007/s00422-018-0755-0) contains supplementary material, which is available to authorized users.

## Introduction

The high interconnectivity of cortical neuronal cells (see e.g., Braitenberg and Schüz 1991) supports the hypothesis that cortical processing is organized in cell assemblies (Hebb 1949; Gerstein et al. 1989), i.e., groups of neurons that act as processing units. Various studies analyzed how cell assemblies may emerge due to different synaptic plasticity rules (e.g., Anderson et al. 1995; Tetzlaff et al. 2015). Active cell assemblies are hypothesized to express temporally coordinated neuronal spiking activity among the member neurons. Different time scales of this coordination have been investigated in numerous theoretical (e.g., Tetzlaff et al. 2007, 2012; Kumar et al. 2010; Diesmann et al. 1999) and experimental studies, across different brain areas (Bair and Koch 1996; Bair et al. 2001; Butts et al. 2007; Price and Born 2010; Murray et al. 2014). Millisecond precision is the fastest of these scales and has been associated with specific mechanisms of neuronal processing. For instance, synchronously incoming spikes to a neuron are known to be more effective in generating an output spike as compared to spikes arriving asynchronously. Building on this fact, neural network models able to process information by exploiting spike synchronization have been proposed (see e.g., Tetzlaff et al. 2015). In the synfire chain model (Abeles 1982, 1991), for example, groups of suitably connected neurons produce, in response to stimulation, synchronous spike volleys that reliably propagate through the cortical network, also in the presence of noise (Diesmann et al. 1999). Activation of a synfire chain may express itself in millisecond-precise spatio-temporal spike patterns (STPs). Their existence in experimental data and their tuning to different behavioral conditions were first shown in Prut et al. (1998). More recent studies extended models to synfire braids or polychronous groups that also build on the fact that neurons mostly reliably emit a spike to synchronous input, however without the requirement that sending neurons are synchronously firing as well (Leen and Shea-Brown 2012; Izhikevich 2006; Bienenstock 1995). Thus, these models predict rather spatio-temporal patterns than spike synchronization.

Numerous studies where two or few neurons were recorded simultaneously found evidence of the repeated occurrence of precisely timed pairs of synchronous spikes in behaviorally relevant contexts across a variety of brain regions (Vaadia et al. 1995; Riehle et al. 1997; Prut and Fetz 1999; Seki and Eggermont 2003; Kohn and Smith 2005; Butts et al. 2007; Pipa and Munk 2011; Shimazaki et al. 2012; Harvey et al. 2013; De Gruijl et al. 2014; Eggermont 2015; Kilavik et al. 2009). In light of the small number of neurons observed in parallel, these studies were blind to possibly existing correlations among larger groups of neurons. Modern electrophysiology enables the simultaneous observation of the spiking activity in the range of 100 or more neurons (Buzsaki 2004; Schwarz et al. 2014). In such massively parallel spike train (MPST) data, the chances to sample from larger groups of neurons engaged in coordinated activity are higher (Nicolelis 2001; Riehle et al. 2013; Hoffman and McNaughton 2002). Even so, though, extracting this information from data of such a size is not trivial. The exponential growth of the number of possible patterns that need to be investigated makes classical direct statistical testing (see e.g., Kass et al. 2014) of each pattern a non-viable approach. Indeed, prohibitive computational resources would be needed to even just count the occurrences of each pattern. Furthermore, the number of statistical tests to be performed would yield excessively many false positives (or false negatives after standard statistical corrections). For all these reasons, the presence and the computational role of millisecond-precise spike correlations, as well as the number of neurons being possibly involved in a cell assembly, are still unclear (Roudi et al. 2009; Ohiorhenuan et al. 2010; Elsayed and Cunningham 2017).

In the 1960s, Gerstein started to develop and apply correlation analysis methods for (few) parallel spike trains. These include the cross-correlation analysis to quantify pairwise correlations (Perkel et al. 1967), the joint peristimulus time histogram for time dependent correlations (JPSTH; Aertsen et al. 1989), the ”snowflake” method for the detection of triplet spatio-temporal patterns (Perkel et al. 1975; Czanner et al. 2005), and an STP detector (Abeles and Gerstein 1988) which was later extended to enable the analysis of a larger number of parallel spike trains (Gerstein and Aertsen 1985; Lindsey et al. 1997; Strangman 1997). The review by Brown et al. (2004) summarized the state of the art of analysis methods and noted the necessity to develop new methods for the analysis of MPST data. Since then, several new methodologies have been introduced to this end. Most algorithms have been designed to enable the identification and categorization of firing rate or spike count correlations on a larger time resolution (e.g., Ganmor et al. 2015; Kelly and Kass 2012; Cunningham and Byron 2014). These would be worth a separate review.

New methods were also developed for the analysis of finer temporal correlations. Among the latter, a number of methods restrict their attention to stimulus-driven responses thereby significantly reducing the number of patterns to be evaluated and the consequent computational and statistical complexity of the problem. For a review of these methods, see Levakova et al. (2015). When a stimulus cannot be clearly identified or isolated from the surrounding environment, or when the stimulus itself is an ongoing internal process rather than an external event, or when recording the stimulus occurrence time is impossible, these methods cannot be applied. For these reasons, more general analysis methods able to deal with the full computational and statistical problems stated above have been recently developed.

This review focuses on such analysis tools, omitting methods that are either not suitable for MPST data, or that reduce their attention to externally driven patterns. We identify in particular two classes of methods for the analysis of temporally precise spike correlations. The first class consists of methods that analyze what we call population correlation, i.e., correlation that manifests at the level of the (full) population of neurons being examined, and does not (necessarily) involve specific cell assemblies. The second class consists of methods designed to identify specific cell assemblies that produce specific types of STPs. In total, we discuss and compare nine methods (four of the first class, five of the second class).

The outline of the paper is as follows. Section 2 introduces different types of correlations in parallel spike trains. Section 3 describes the methods for correlation analysis considered here, clarifying their assumptions and for which type of correlation they were designed to detect. Section 4 compares the considered methods in terms of their sensitivity to the different correlation models and discusses their ability to reconstruct (entirely or partially) those correlation structures. A perspective on new research avenues that these methods open is given in Sect. 5.

## Models for parallel correlated spike trains

Temporal coding has been associated with different (but not necessarily incompatible) forms of spike correlation at fine temporal scale, i.e., with ms precision. These can range from synchronization of always different cell groups, to spike sequences from specific neurons in a specific temporal order, to sequences of synchronous activity. Each method considered in this paper was designed to determine the presence of one such correlation structure in MPST data. Hence, it is first necessary to introduce the respective correlation models and to highlight their similarities and differences. This section presents five different types of fine temporally correlated spiking activities that have been associated to mechanisms of temporal coding in the literature, either in theoretical or in experimental studies. Additionally to the heuristic description provided in this section, in the Supplementary Material 6.1, we define formally a Point Process framework that can be used to model and generate artificial data for the different correlation structures here introduced.

### Population synchronization

Population synchronization refers to spiking activity where some of (or all) the neurons observed emit synchronous spikes, repeatedly over time. The neurons involved are not hypothesized to be always the same, although they may. For this reason, methods designed to detect the presence of synchronization at the population level do not need to look for and to assess the statistical significance of a multitude of different spike patterns.

**Fig. 1:**
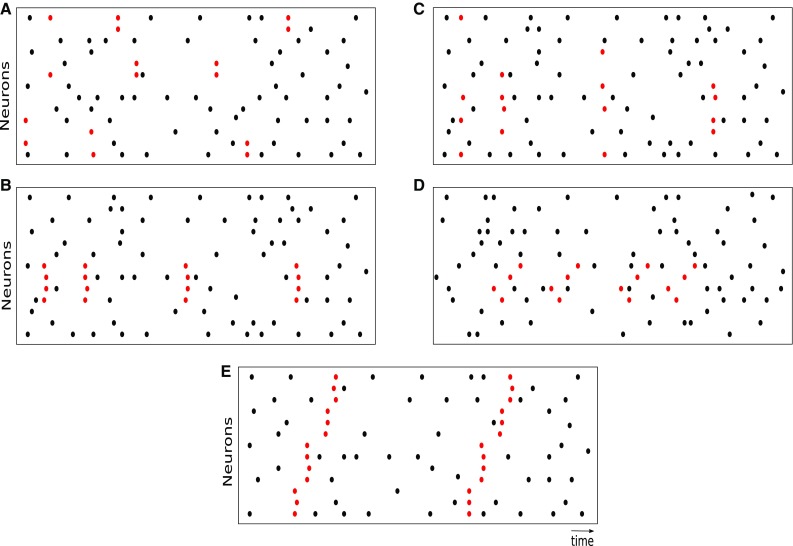
Raster plots of different correlation types. Each panel shows the spiking activity of parallel spike trains (one neuron per row) over time (horizontal axis). Each dot represents a spike; the red dots in particular represent spike belonging to a spike pattern. Different panels refer to different forms of temporal spike correlation. **a** Pairwise correlation model. The population contains 6 pairs of synchronized neurons (the latter indexed from bottom to top): $(1,2),\ (1,3),\ (2,4),\ (8,9),\ (8,14),\ (13,14)$. **b** Synchronous spike patterns. Neurons 4, 5, 6, 7 are repeatedly involved in the pattern. **c** Differently from the spike patterns in panel **a**, the neurons involved in each synchronous event are randomly selected and change from one event to the next.**d** Spatio-temporal patterns. The red spikes correspond to occurrences of an STP. The neurons involved in the patterns are 4, 5, 6, 7, as in panel **a**, but their spikes occur now in a fixed temporal succession with fixed delays. **e** Sequences of synchronous spike events. Two occurrences of the same SSE are shown. Here, all observed neurons are involved, and groups of 4-4-4-3 synchronously firing neurons fire in short succession

This fact per se does not exclude the presence of specific cell assemblies in the data being recorded. A neural network model that contains cell assemblies and may or may not produce repeated spike patterns, depending on the model parameters, is the synfire chain. A synfire chain is a network with a high convergent and divergent connectivity from one layer of neurons to the next (Abeles 1991). The network exhibits synchronous spiking activity that, triggered by stimulation of the first layer, propagates through the next layers. The propagation is robust to noise (Diesmann et al. 1999). However, the latter study also showed that the composition of the active neurons may vary at each run, depending on the connectivity and its strength. If so, recordings from neurons in the same layer would contain different, although possibly overlapping, synchronous spike patterns (see Fig. 1c). From a statistical perspective, a probabilistic model of parallel spike trains able to generate different but overlapping synchronous spike patterns and often used for method validation to generate ground truth data, is the multiple interaction model (Kuhn et al. 2002, 2003).

### Pairwise synchronization

In the 1960s researchers first started to look into correlations between spike trains with the idea that correlated neurons reflect functional correlation. Gerstein and Clark (1964) and Perkel et al. (1967) developed the cross-correlation analysis to detect correlations between two parallel spike trains beyond trivial effects like stimulus dependent rate increase. Many other studies then followed, a large collection of which is found in the book by Eggermont (1990).

In pairwise synchronization, pairs of neurons synchronize their spikes independent of each other. Thus, higher-order correlations are absent. Patterns of size 3 or larger are still possible, however, only as the result of chance simultaneous spike emissions from individual neurons or neuron pairs. This type of spiking activity is shown in Fig. 1a.

In studies concerned with the analysis of spike correlations, there was and there still is a focus on pairwise analysis in the field (Riehle et al. 1997; Kilavik et al. 2009; Vaadia et al. 1995; Zandvakili and Kohn 2015). The reason is not that the theory would predict pairwise correlations only (see, e.g., Abeles 1982), but rather the simplicity of such analyses over that of higher-order correlations. Nevertheless, the study of purely pairwise correlations may reveal, in MPST data, interesting dependence structures that hint to larger interacting groups of neurons, cross-area interactions or spatial interactions. Some of the methods reviewed in this paper analyze pairwise correlations for statistical significance, and then group them into larger groups of interacting neurons.

### Synchronous spike patterns

A neuron receiving synchronous synaptic inputs is more likely to emit a spike than asynchronously arriving inputs, as predicted by theory (Abeles 1982; König et al. 1996; Fries 2005; Schultze-Kraft et al. 2013) and shown in experiments (Ashida et al. 2016). This observation led to hypothesize that neurons synchronize their activities beyond pairs. To investigate this hypothesis in real data by statistical testing, as well as to generate synthetic data for method validation, probabilistic models of parallel spike trains including higher-than-pairwise synchronization were formulated. Two examples are the single interaction model by Kuhn et al. (2002), and the maximum entropy model by Schneidman et al. (2003). A realization of the single interaction process where a synchronous spike pattern has multiple occurrences is shown in Fig. 1b.

### Spatio-temporal patterns 

Spike synchrony can be generalized by adding a temporal dimension to the correlation: the neurons involved in the coordinated activity do not necessarily spike synchronously, but in specific temporal sequences with fixed (up to a given precision) delays between consecutive spikes (see Fig. 1d). This type of activity is generally referred to as a spatio-temporal pattern (STP; Prut et al. 1998).

STPs may be the results of variability of conduction delays observed in cortical network (see e.g., Swadlow 1994) and may arise in different network models. For instance, a synfire chain produced STPs where neurons in the same layer of the chain fire synchronously, while neurons belonging to different layers fire at fixed delays. If one would record only one neuron per layer, the STP would reduce to asynchronous spikes with fixed delays. Another model that generates STPs is the synfire braid (Bienenstock 1995), also called polychrony model (Izhikevich 2006). It is a generalization of the synfire chain, in which spikes produced in one layer arrive at the next layer at different times due to different propagation delays. Various methods have been developed to extract STPs from a small number of parallel spike trains (Dayhoff and Gerstein 1983; Prut et al. 1998; Abeles and Gerstein 1988), and these methods retrieved statistically significant STPs in experimental data (see, e.g., Prut et al. 1998).

### Sequences of synchronous spike events

A specific type of temporal correlation that features spike synchronization and temporal propagation is represented by sequences of synchronous events (SSEs). These consist of multiple synchronous events, each involving a specific group of neurons, occurring at a fixed temporal delay one after another. Parallel recordings from multiple layers of an active synfire chain would for instance exhibit such spike patterns (Schrader et al. 2008; Gerstein et al. 2012). The sets of neurons involved in different synchronous events may or may not overlap. A realization of one specific SSE occurring two times is shown in Fig. 1e.

## Higher-order correlation analysis methods

In this section, we summarize existing statistical methods for the detection of higher-order correlations in MPST data. We give a short description of these methods, highlighting their features and limitations, in particular with regard to how they deal with different properties of uncorrelated background activity in the data. For details, we refer to the original publications (Table 1).

Generally, two classes of methods can be distinguished, which investigate different aspects of spike correlation. The first class aims to identify the correlation order (number of neurons involved) rather than the identity of the neurons involved. Thus, each correlated event may involve a random subset of neurons or may be composed of a specific, always identical group. We refer to the correlation type underlying this analysis class as *population synchronization*. The other class of analysis methods assumes a correlation model in which the correlated neurons form stereotypical synchronous spike events or temporal sequences of spikes. We refer to these events as *spike patterns. *The aim of these methods is to retrieve the neuronal composition and the occurrence times of the spike patterns.

**Table 1:** Table of analysis methods, their assumptions, and related references

Method	Target correlations	Null model	Alternative model	References
Population synchronization				
CD	Population synchronization	Independent spike trains	Population synchronization	Grün et al. (2008) and Louis et al. (2010a)
CUBIC	Population synchronization	Population synchronization of order $\xi$	Population synchronization of order $\xi +1$	Staude et al. (2010a) and Staude et al. (2010b)
PUE	Population synchronization	Population synchronization of order $\xi$	Population synchronization of order $\xi +1$	Rostami (2017)
CII	Population synchronization	Maximum entropy model of order $\xi$	Maximum entropy model of order $\xi +1$	Schneidman et al. (2003) and Schneidman et al. (2006)
Spike patterns				
MEM	Synchronous spike patterns	Maximum entropy model of order $\xi$	Synchronous spike patterns	Schneidman et al. (2003), Schneidman et al. (2006), Shimazaki et al. (2012) and Kelly and Kass (2012)
GIC	Synchronous spike patterns	Independent	Pairwise synchrony	Berger et al. (2010)
SPADE	Synchronous and spatio-temporal spike patterns	Independent	Synchronous pattern, Spatio-temporal patterns	Borgelt (2012), Torre et al. (2013) and Quaglio et al. (2017)
CAD	Synchronous and spatio-temporal spike patterns	Poisson independent	Synchronous pattern, Spatio-temporal patterns	Russo and Durstewitz (2017)
ASSET	Sequences of synchronous events	Poisson independent	SSEs	Schrader et al. (2008), Gerstein et al. (2012) and Torre et al. (2016a)

The table summarizes the methods that we discuss here and their assumed data models (column 2 from left, all introduced in Sect. 2). Columns 3 and 4 describe the assumed null and the alternative hypothesis, respectively. Column 5 lists the publications in which each method has been introduced or further developed

### Methods to detect population synchronization

One of the challenges in the statistical assessment of synchronous spike events in MPST data is posed by the exponential growth of the number of possible patterns with the number of neurons being considered. However, this problem can be simplified if the research interest lies solely on assessing the presence and the order of excess (i.e., above-chance) synchronization, without resolving the specific neuron identities involved. Also, the data may contain patterns of synchronous spikes that change their neuronal composition each time, so that the correlation is distributed possibly across the full population being observed. We refer to synchrony which does not (or which is not assumed to) involve specific subgroups of neurons in the observed population as population synchronization.

Most methods for population synchronization analysis reduce the spike data to the number of active neurons (i.e., spikes) observed at any time bin. A spike train is fully described by its spike times and, given a time discretization in small temporal bins, we can define the population histogram as the count of spikes that occurred in the same time bin. The maximum possible count of the histogram is thus the number *N* of neurons. The first three methods presented here are based on statistics derived from the population histogram. They were developed in succession, each to overcome the limitations of the previous one. The first method, the Complexity Distribution (CD) analysis (Grün et al. 2008), proposes a simple statistical approach purely based on the distribution of the entries of the population histogram. It compares such an empirically derived distribution to that expected from neurons firing independently to determine the presence of excess synchronization. The second method, the CUmulant-Based Inference of Correlation (CuBIC, Staude et al. 2010a), derives the null distribution analytically under more specific assumptions about the data, and infers the minimum correlation order existent in the data. The third method, the Population Unitary Event (PUE, Rostami 2017) analysis, works under the same assumptions as CuBIC, but uses a different test statistic which enhances the statistical power of the test, thereby requiring samples of smaller size for a correct identification of excess synchrony and thus also enabling a time-resolved analysis.

The fourth method, called here the correlation information index (CII), is an approach originally suggested by Schneidman et al. (2006) as a way to condense the information delivered by maximum entropy models built on parallel spike train data to a single scalar. The method accounts for the neuronal identity of each spike in the observed synchronous patterns and builds a full probabilistic model of those. This model is used to obtain a single scalar, the CII, that quantifies the amount of surplus of information contained in the data which is delivered by correlations of a given order.

#### Complexity distribution (CD)

The value taken by each entry in the population histogram is called the bin complexity. Each synchronous spike event increases the empirical complexity in the bin of it’s occurrence as compared to the scenario of independent spiking. Therefore, it also increases the value of the empirical complexity distribution at that complexity value. Grün et al. (2008) developed a method that tests for spike train independence based on the difference between the empirical complexity distribution and the null distribution. Excess synchrony causes the difference between the two distributions to have a positive bump at larger complexities. Due to the conservation of the total probability mass, a negative bump appears at lower complexities. Depending on the assumptions about the spiking behavior of each neuron, the null distribution may be available analytically (e.g., by assuming that the spike trains are stationary Poisson processes) or may be approximated by Monte Carlo surrogate techniques (see Grün 2009; Louis et al. 2010a, c), and will in general depend on the statistics of each spike train as well as on the chosen bin size.

**Fig. 2:**
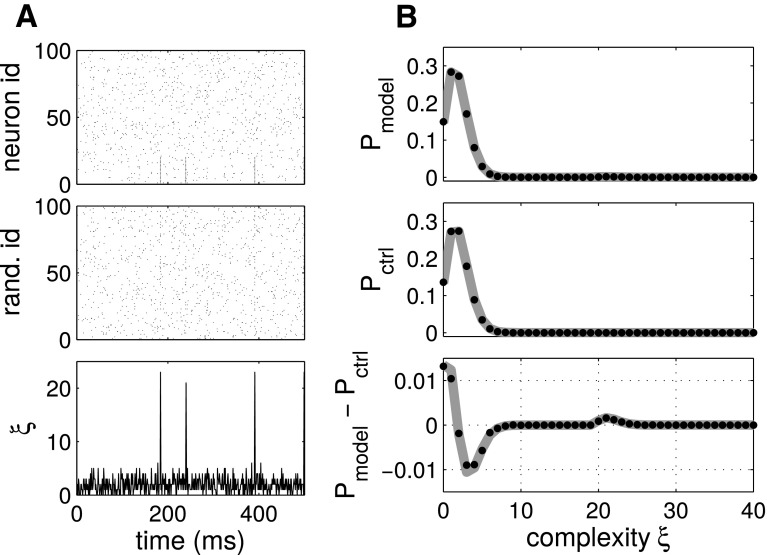
Complexity distribution based correlation identification. **a** *Top* Parallel spike trains comprising a synchronous spike events among the first 20 neurons, firing in synchrony with a rate of $\lambda _{c}=5$ 1/s, plus 80 independent neurons. *Middle* randomization of the neuron ids (vertical axis) of the top panel. *Bottom* population histogram of the data (bin width: $w=1\,\text {{ms}}$). **b** *Top* Complexity distribution of the data in **a**. *Middle* null distribution obtained analytically (solid line) or by surrogates through spike time randomization in time (dots). *Bottom* difference between the observed and the null complexity distributions (Reproduced with permission from Grün et al. 2008)

An example of artificial test data is illustrated in Fig. 2, modified from Grün et al. (2008). Panel A, top, shows data from a stochastic simulation of 100 parallel spike trains, 80 of which are independent Poisson. The first 20 neurons exhibit, in addition to independent spiking activity, also synchronous firing events. The synchronous events are hardly visible by eye in the raster plots if the neuron ids on the vertical axis are sorted randomly (Panel A, middle), but can be retrieved in the population histogram (Panel A, bottom; bin size: $1\,\text {{ms}}$), although with a loss of information about the involved neurons. Panel B shows the empirical complexity distribution (top), the null distribution computed by randomizing the spike times of each neuron (middle), and the difference between the two distributions (bottom). The latter contains a visible bump centered at complexity ${\xi }=22$. Importantly, the bump is right-skewed and is centered to the right of the true synchronization order $\xi =20$. The reason for the offset in the peak is that the inserted synchronous events of fixed size $\xi$ overlap with background activity from the other neurons, resulting in a higher total complexity. The bin width *w* determines the statistics of the random component of the total count.

Under the assumption that all spike trains are Poisson processes with identical firing rates, the null distribution can be computed analytically based on combinations of Binomial distributions (Grün et al. 2008; Fig. 2b, solid). Otherwise, it can be computed by surrogates, e.g., by spike time randomization (Fig. 2b, dots). Confidence intervals are computed analogously, and allow to accept or reject the null hypothesis of independence (Louis et al. 2010a). Varying the bin size enables to determine the temporal jitter inherent to the synchronous events (for details, see Louis et al. 2010c).

#### CUmulant-Based Inference of Correlation (CuBIC)

The complexity distribution method discussed above visualizes correlations among parallel spike trains. The CUmulant-Based Inference of Correlation (CuBIC; Staude et al. 2010a) advances this technique by relaxing the hypothesis of independence and testing for the presence of correlations of progressively higher order, given those of lower order observed in the data.

**Fig. 3:**
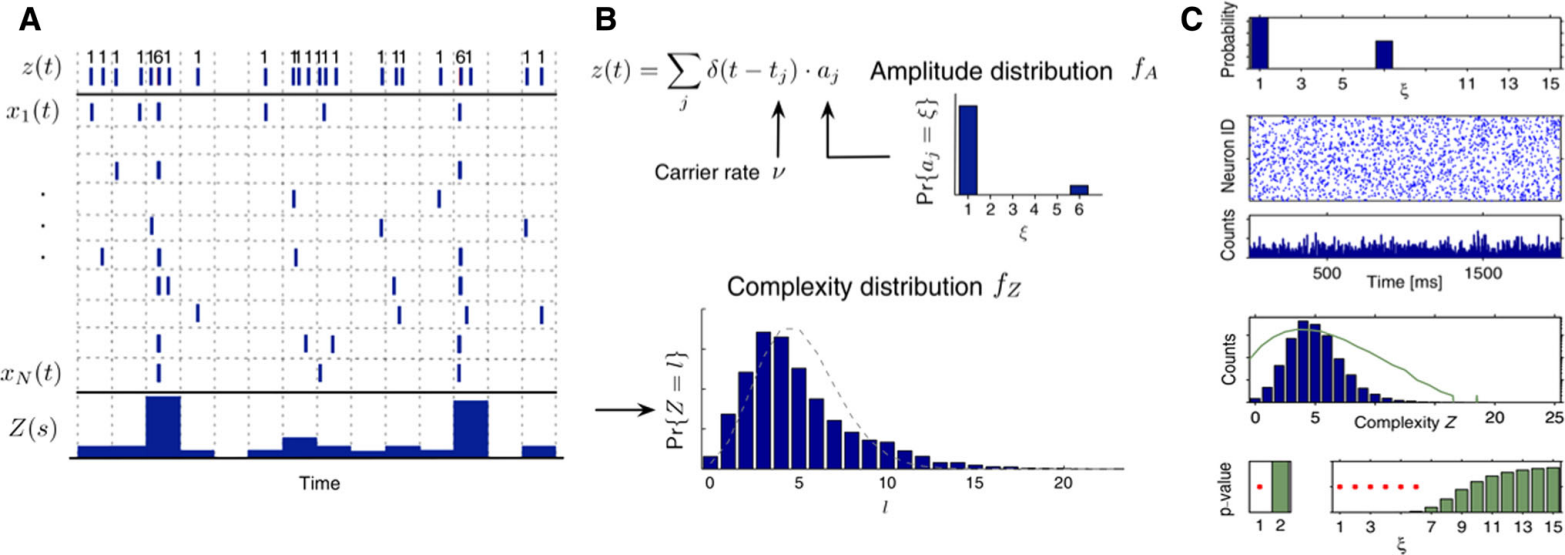
CuBIC analysis. **a**, **b** Illustration of the generation of correlated parallel spike trains using a marked point process. This process is the null model used to test $\xi =6$. Spikes are assumed to be copied from a hidden process *z*(*t*), (**a**, top) consisting of spike times $t_{i}$ drawn from a Poisson process and associated labels $a_{j}$ drawn from the amplitude distribution $f_{A}$ (**b**, top). Each spike $t_{i}$ in the hidden process is copied into $a_{j}$ spike trains, randomly selected each time from the full population $x_{1},\ldots ,x_{N}$. The population histogram *Z* (**a**, bottom) is computed by segmenting the time axis into consecutive bins of a few ms. The complexity distribution $f_{Z}$ (**b**, bottom panel) is derived from *Z*.**c** Application of CuBIC to simulated correlated data. The figure shows, from top to bottom: the amplitude distribution used to generate the correlated data, the raster plot of the generated data, the derived population histogram, the empirical complexity distribution (blue) and its logarithmic transform (green), the test p values for different orders of correlation tested by CuBIC (Reproduced with permission from Staude et al. 2010a)

CuBIC comprises the following steps. Starting from $\xi =1$ (spike train independence), it assesses whether peaks in the complexity distribution of the data could be explained entirely by assuming correlations of order at most $\xi$. If that is not the case, the method accepts the alternative hypothesis that correlations of order $\xi +1$ or higher must exist. It then tests for correlations of order $\xi +1$ against those of order $\xi +2$ or higher, and so on. The procedure stops as soon as a value $\hat{\xi }$ is accepted. $\hat{\xi }$ is interpreted as the minimum order of population synchronization that has to be assumed to explain the observed amount of synchronous events in the data. The sequence $(p_{1},\dots ,p_{\hat{\xi }})$ of test p values is guaranteed to increase, because synchronous events of higher complexities correspond to higher expected correlations, and thus eventually exceeds the selected significance threshold $\alpha$, terminating the procedure (see last row of panel C in Fig. 3).

The test *p* value is obtained analytically in the limit of a large number *L* of i.i.d. observations (time bins), and by assuming that all spike trains are Poisson processes. In particular, the case $\xi =1$ corresponds to the assumption that the spike trains are independent. The case $\xi >1$ corresponds to the assumption that up to $\xi$ neurons synchronize their spikes with positive probability. The probability of synchronous events of a given size is modeled by the so-called amplitude distribution (see panel b in Fig. 3). The analytical formulation makes CuBIC computationally inexpensive, but requires *L* to be large enough (according to Staude et al. 2010a, $L\ge 10^{5}$ bins) to get reliable results. The analysis is therefore limited to applications of relatively long and stationary data. The length of the data required does not enable the method to reveal changes of the correlation order over time. While the original publication developed the method for stationary data, generalizations had been later on provided for populations of spike trains with specific firing rate distributions, such as Gamma or uniform distributions (Staude et al. 2010b) or non-stationary processes (Reimer et al. 2012).

#### Population unitary event (PUE)

**Fig. 4:**
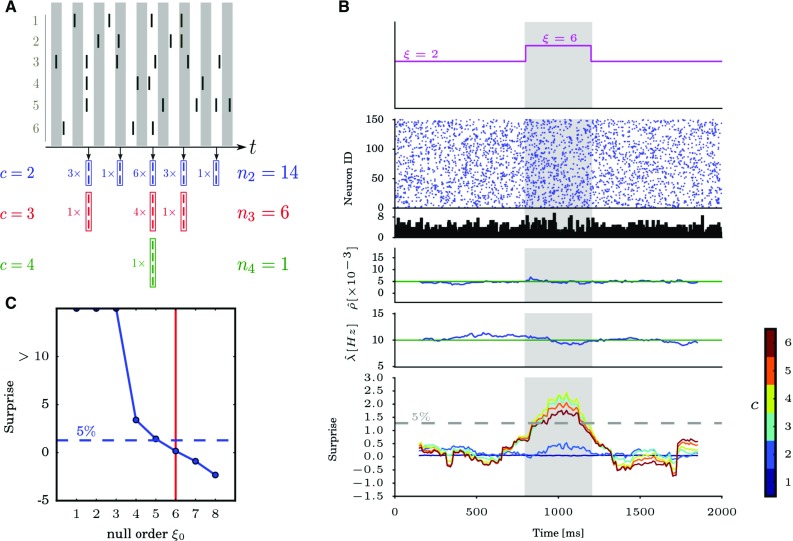
Population unitary event analysis. **a** Top: raster plot of 6 neurons firing over time. Bottom: illustrative example of the test statistic $n_{c}$, showing for given values of *c* ($c=2$ blue, $c=3$ red, $c=4$ green) and for each bin of the population histogram containing $Z_{k}\ge 2$ spikes, the number $$\left( \begin{array}{l}Z_{k}\\ {c}\\ \end{array}\right) $$ of patterns of size *c* that can be extracted from the $Z_{k}$ spikes. **b** Time-resolved PUE analysis applied to simulated data. The simulated data consist of $N=150$ parallel spike trains with duration $T=2000\,\text {{ms}}$, generated as a realization of a correlated Poisson process of order $\xi =6$ in the time window $[800\,\text {{ms}},1200\,\text {{ms}}]$ (indicated in gray), and of order $\xi =2$ elsewhere. The firing rate of each individual spike train is set to $10\,\text {Hz}$ and the pairwise correlation coefficient to 0.005. The data are analyzed with PUE varying the hyperparameter *c* from 1 to 6. From top to bottom: Time course of synchrony order $\xi$, raster plot of the data, population histogram ($1\,\text {{ms}}$ bin size), cross-neuron average of empirical pairwise correlation coefficients calculated over a $300\,\text {{ms}}$ sliding window, average firing rate estimated over the same sliding window, and surprise measure of the PUE statistic using different values of the parameter *c*. The surprise is calculated for each time window with null order $\xi _{0}=2$. Different colors correspond to different values of *c*. The gray dashed line indicates the $5\%$ significance level. **c** Estimation of the synchronization order $\xi$ in the central analysis window (highlighted in gray in panel B), for a null order $\xi _{0}$ increasing from 1 to 8. The data are obtained by concatenating 15 model realizations generated as explained in B, and mimicking identically distributed experimental trials. The blue dashed line indicates the $5\%$ significance level and the vertical red line shows the true synchronization order $\xi =6$ (Reproduced with permission from Rostami 2017)

As mentioned in the previous section, CuBIC is limited in its application to long stretches of stationary data. However, experimental results regarding pairwise correlation analysis using the unitary events analysis method (Riehle et al. 1997; Kilavik et al. 2009) revealed that excess synchronization may appear dynamically and related to behavior. Thus, time-resolved analysis methods for detecting higher-order correlation are required. The population unitary event (PUE) analysis method is designed to enable that. The test statistic of PUE is the number of synchronous spike events of a given size *c* observed in the data, which is extracted from the population histogram. For bins containing spike counts *Z*, we consider the total number of possible constellations of *c* from *Z*, thus $$(\begin{array}{l}Z_{k}\\ {c}\\ \end{array})$$ per bin *k*. Thus, in a total of *L* bins, we derive the number of synchronous spike events of size *c* according to $$n_{c}=\sum _{k=1}^{L}\left( \begin{array}{c} Z_{k}\\ c \end{array}\right) =\sum _{k=1}^{L}\frac{Z_{k}!}{c!(Z_{k}-c)!}.$$ For example, as shown in Fig. 4a, $n_{2}$, $n_{3}$ and $n_{4}$ are the total number of distinct pairwise, triple-wise and quadruple-wise synchronous spike events present in the data.

The PUE analysis exploits the following framework for testing the significance of the empirical test statistic observed in the empirical data. The null hypothesis of the PUE method is defined by a presumed order of synchrony among the spike trains, i.e., the null order $\xi _{0}$, and *assumes that the order of synchrony among the given spike trains is at most the null order* $\xi _{0}$. The null distribution and the associated test p value are computed numerically by a Monte Carlo simulation by realizing a marked Poisson process (see Ehm et al. 2007; Staude et al. 2010a), and see the Supplementary Material in Sec. 6 used to model a multidimensional correlated Poisson process (as also assumed and introduced in CuBIC, Sect. 3.1.2). The parameters for the null model are adapted by the firing rate and the pairwise correlation parameters extracted from the data (see details in Rostami 2017; Staude et al. 2010a).

The analysis can be performed in a time-resolved fashion by sliding a window through the data in steps of a few time bins, and by analyzing each time window separately. As shown in Fig. 4b, the surprise measure, defined as a logarithmic transformation of the *p* value (Palm 1981), becomes significant when the analysis window overlaps with the synchronization period. This enables a time-resolved analysis which is able to discover changes in the correlation order over time.

When multiple experimental trials are available, the PUE method may pool data from different trials to achieve increased statistical power, under the assumption of cross-trial stationarity. Figure 4c shows an example where the order of synchrony is inferred by PUE using all 15 trials. By computing the surprise as a function of the null order $\xi _{0}$, the estimate $\hat{\xi }$ of true order of synchrony in the data can be obtained as the lowest value of the null order $\xi _{0}$ for which the surprise measure is not significant. PUE has higher statistical power (and therefore needs less evidence) than CuBIC to detect existing correlations in data.

#### Correlation information index (CII) 

Maximum entropy models (MEMs) have been introduced to evaluate the occurrence probability of each synchronous spike pattern (seen as a binary sequence of on/off states) given the observed firing rates, pairwise correlations, and possibly higher-order moments of a population of observed neurons. Once a maximum entropy distribution accounting for all and only the observed correlations up to a given order $\xi$ is inferred from data (see Sect. 3.2.1 for more details), the amount of information delivered by such correlations can be quantified as follows.

The larger is the order $\xi$ of the moments one accounts for to construct the maximum entropy distribution, the smaller is the total entropy $\mathcal {H}_{\xi }$ of the maximum entropy distribution (that is, its uncertainty). At one extreme ($\xi =1$, only average firing rates being considered), one gets the uniform distribution, where the probability of a state is proportional to the product of the firing rates of the “on” neurons. At the other extreme ($\xi =N$, where *N* is the number of neurons) one gets the empirical distribution. The entropy $\mathcal {H}_{\xi }$ of the maximum entropy distribution constrained on all moments up to order $\xi$ decreases, for $\xi$ increasing from $\xi =1$ to $\xi =N$, from $\mathcal {H}_{1}$ to $\mathcal {H}_{N}$. The difference $\mathcal {H}_{1}-\mathcal {H}_{\xi }$ quantifies the reduction of the entropy (i.e., of the uncertainty about all possible states) due to the knowledge of all correlations of order 2 to $\xi$, that is, the amount of information conveyed by those correlations. The difference $\mathcal {H}_{1}-\mathcal {H}_{N}$ quantifies the total information delivered by correlations of any order. Thus, the ratio

$$\begin{aligned} R_{\xi }=\frac{\mathcal {H}_{1}-\mathcal {H}_{\xi }}{\mathcal {H}_{1} -\mathcal {H}_{N}} \end{aligned}$$

**Fig. 5:**
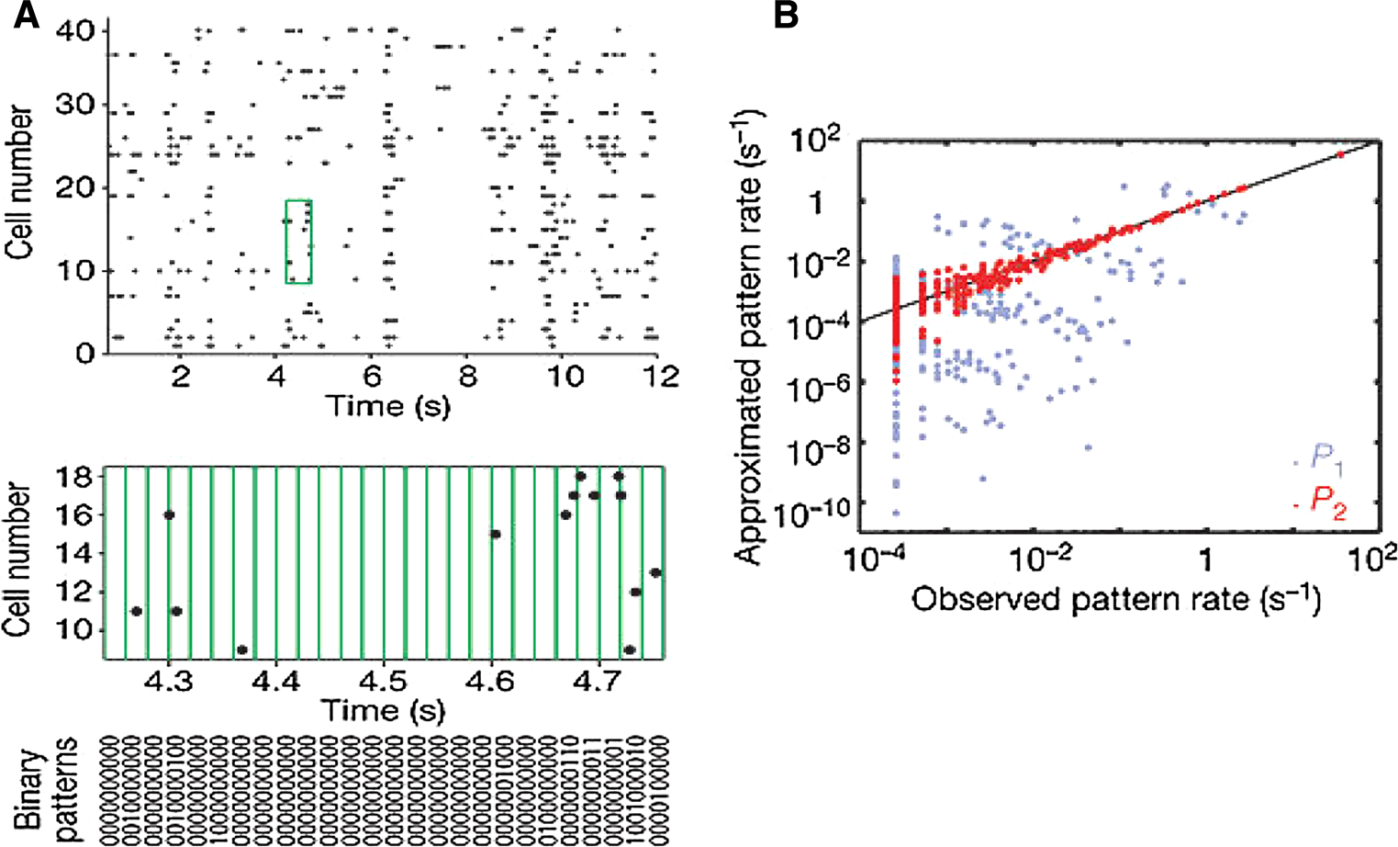
Maximum entropy models. **a** A segment of the simultaneous responses of 40 ganglion cells in the salamander retina to a natural movie clip (top panel). Discretization of parallel spike trains into binary patterns is shown below (green). The binary vectors describe the joint activity patterns of the cells at a given time point. For clarity, 10 out of 40 cells are shown (bottom panel). **b** Using the same group of 10 cells from panel **a**, the rate of occurrence of each firing pattern predicted from a maximum entropy model $P_{2}$ that takes into account all pairwise correlations is plotted against the pattern rate measured in the recorded data (red dots). For comparison, the independent model $P_{1}$ is also plotted in gray. The black line indicates equality (Reproduced with permission from Schneidman et al. 2006)

characterizes the portion of the total correlation information delivered by correlations of order 2 to $\xi$. This measure is called the correlation information index (CII). $R_{2}$ was suggested by Schneidman et al. (2003) to assess whether or not triple- or higher-order correlations play a role in information processing in the nervous system. Schneidman et al. (2006), Shlens et al. (2006) and Tang et al. (2008), among others, applied this measure to data from the retina and from various cortical areas, reporting values ranging from 0.85 to over 0.95. Based on these high values, they concluded that higher-order correlations were negligible in the examined data. It should be noted, nevertheless, that even for extremely high values of $R_{2}$ ($R_{2}>0.99$), highly statistically significant spike patterns of 3 or more neurons may be present in the data (Torre 2016). In addition, Roudi et al. (2009) showed in a theoretical study that conclusions obtained from MEMs built on data from few neurons cannot be extrapolated to larger samples of parallel spike data. Nevertheless, this approach may be helpful to quantify the amount of information present in parallel spike train data which is delivered by correlations of a certain order.

### Methods for spike pattern detection

The second group of methods covered in this review is designed to detect groups of neurons involved in millisecond-precise spiking patterns. These methods achieve this goal by detecting spike patterns that repeat sufficiently many times to be classified as non-chance patterns. Non-chance patterns are considered a signature of assembly activation (Abeles 1991), and have been associated with behavior in several experimental studies (e.g., Prut et al. 1998). The large number of possible patterns in large scale recordings often poses non-trivial computational and statistical problems. To get a flavor of this problem, consider a population of *N* neurons recorded in parallel. These neurons may organize their activity in up to $2^{N}$ different patterns of synchronous spikes, which is close to $10^{30}$ for $N=100$, as regularly available in modern extracellular recordings. This number increases by orders of magnitude if arbitrary STPs, and not only synchronous events, are considered. Without any previous knowledge about the neurons possibly involved in the correlation, a blind search for patterns occurring more than expected under some null hypothesis has to be performed, accounting for all these possibilities. The computational burden may be excessive (even allocation of the occurrence counts of all possible patterns to memory may be impossible). Besides, testing all patterns individually for statistical significance would yield insurmountable multiple testing issues. Finally, the amount of data needed to collect adequate statistical evidence would be immense, and most likely unavailable. The methods considered here have been developed to address these issues. We specifically restrict our attention to methods that can be applied to large scale recordings, and whose ability to discover existing patterns has been demonstrated on simulated data. Also, we disregard those methods that search only for patterns temporally locked to some stimulation. A recent review of the latter can be found in Levakova et al. (2015).

**Fig. 6:**
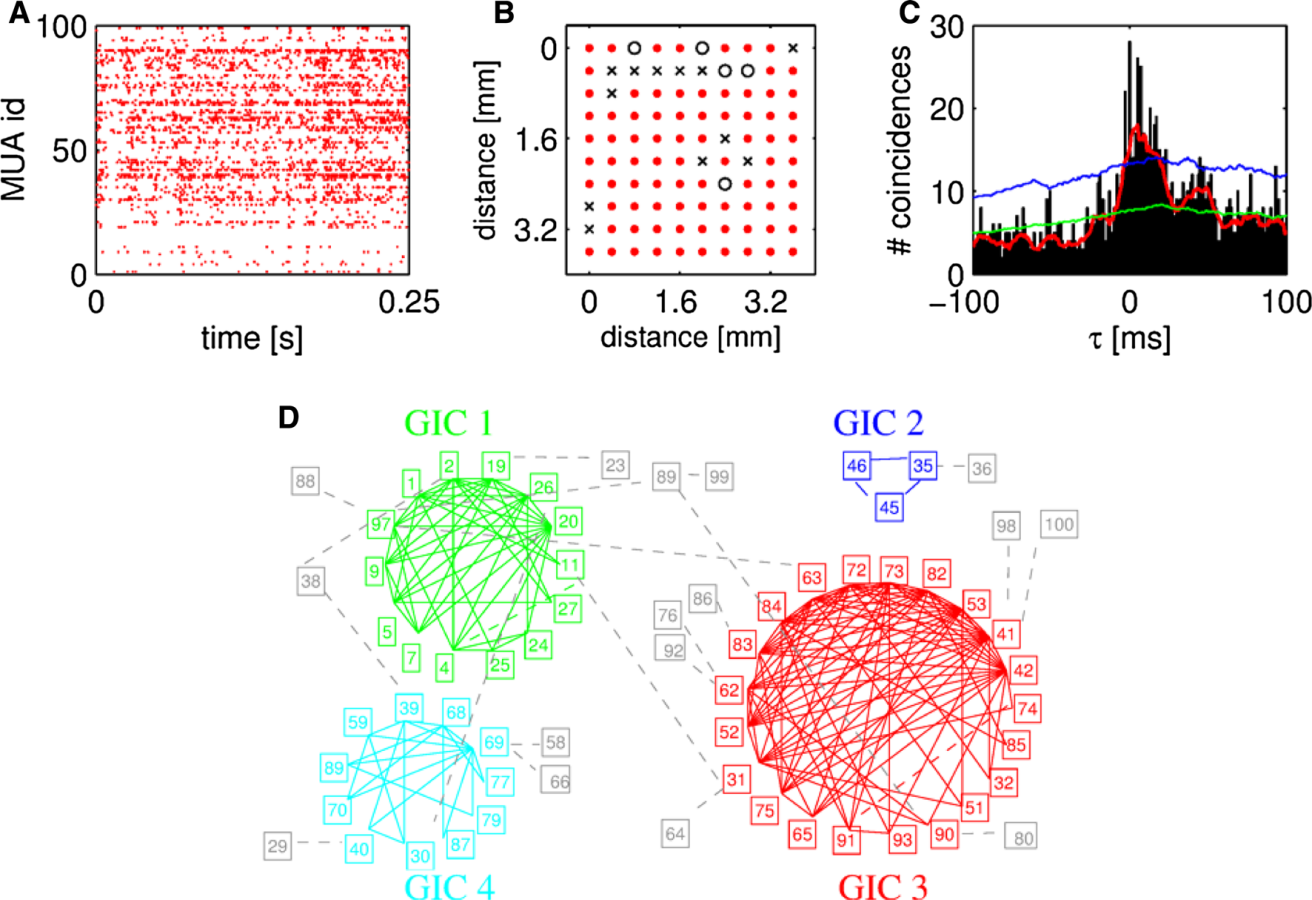
Cliques of pairwise correlated spike data. **a** Raster display of 84 simultaneously recorded multi-unit (MUA) spike trains, i.e., spikes of the same train were not sorted into single neurons. Some of the electrodes did not record any data, thus the corresponding line is empty. **b** Arrangement of the 100 electrode recording array (Utah array). Empty circles mark electrodes that were not connected, electrodes marked with a cross did not work. The rest (red dots) indicate working electrodes, from which the data in panel A were recorded from. The non-diagonal next electrode distance was $L=400\,\mu m$. **c** Example CCH of two multi-unit spike trains. Black: cross-correlation of the recorded data, bin width: 1 ms, red line: smoothed CCH (rectangular kernel of 10 ms width). The green line shows the bin-wise average CCHs of the surrogate data (100 repetitions) generated by spike dithering with $\pm 35\,\text {{ms}}$, and smoothed as the original CCH. It represents the expected CCH under the null hypothesis. The blue line indicates $+$ 2 std of the bin-wise entries of the surrogate CCHs. A pair of MUAs is considered significantly correlated if the smoothed original CCH (red) exceeded at or around $\tau =0$ this significance level. **d** Clusters of cliques of significantly correlated pairs of MUAs. A significantly correlated MUA pair is represented by two nodes (each MUA id is shown in the respective rectangle). Groups of 3 or more all-to-all correlated MUAs are clustered into cliques. Cliques sharing at least one node are further combined into a group of intracorrelated cliques (GIC). The resulting four clusters are marked in different colors (red, green, cyan and blue). MUAs not fulfilling these criteria are marked by gray squares, connected by dashed lines to the other MUA they are correlated with (Reproduced with permission from Berger et al. 2007)

#### Maximum entropy models (MEM)

As mentioned already in Sect. 3.1.4, MEMs provide the possibility to assess the likelihood of specific spike patterns based not only on the average neuronal firing rates, but also on the observed second and higher-order correlations. As shown in Fig. 5, a MEM of order $\xi$ converts spike trains to binary sequences by binning, computes the average zero-lag correlations up to order $\xi$ (the vector of average firing rates, the matrix of second order correlation coefficients, the tensor of third order correlations, and so on), and then provides an analytical estimate of the p value of any spike pattern under these constraints (and under the additional assumption that the spike trains are Poisson). A joint distribution of *N* binary states (on/off neurons) is fully specified if and only if all multivariate moments up to order *N* are given. MEMs specify only the correlations up to an order $\xi <N$, and then determine the maximum entropy (the least assertive) distribution among all the distributions compatible with the given constraints (see, Jaynes 1957).

By constraining the distribution to correlations up to a given order, the presence of “genuine” higher-order correlations (that is, of correlations that are not expected based solely on the observed lower order correlations) can be ascertained. The analytical treatment provides an efficient way to analyze data from relatively large parallel recordings. This methodology has been used in a number of studies to search for statistically significant synchronous spike patterns, constraining on the observed average neuronal firing rates and average pairwise correlations ($\xi =2$) (see, e.g., Schneidman et al. 2006; Tkacik et al. 2006; Tang et al. 2008). Shimazaki et al. (2012) extended the method to account for time varying interactions. Kass et al. (2011) and Kelly and Kass (2012) incorporated in the null hypothesis history effects that make the spike trains deviate from the Poisson assumption.

Despite these efforts, a number of short-comings limits the applicability of MEMs to MPST data. First, the maximum entropy distribution of a large number of neurons is computationally demanding to evaluate due to the large number of parameters to be determined. This is the more so if non-stationarities are taken into account, which is necessary in most applications to avoid biased statistics. Second, evaluating the p value of each pattern individually leads in MPST data to multiple testing issues, resulting in excessive false positives (or false negatives after standard statistical corrections like, e.g., the Bonferroni correction). Third, Rostami et al. (2017) studied in detail the aptness of MEMs in application to MPST data and showed that MEMs predict a bimodal distribution for the population-averaged activity, when it is applied to typical experimental recordings of 150 or more neurons. Thus the MEM distribution is not uniquely predicted, but switches between different states of activities for long data sets. For these reasons, the MEM model does not easily scale to data of large populations of neurons, but can be accounted for by an extended model (Rostami et al. 2017). Nevertheless, MEMs provide valuable tool to analyze genuine higher-order synchronous events.

#### Neuronal cliques and groups of intracorrelated cliques (GIC)

A first approach to analyze MPST data for the presence of cell assemblies of possibly large size involved in correlated activity is the Groups of Intracorrelated Cliques (GIC) analysis, developed by Berger et al. (2007). The method first determines pairs of correlated neurons using the cross-correlation histogram (CCH; Perkel et al. 1967), then groups overlapping pairs into larger groups which possibly indicate higher-order interactions.

The CCH between a reference and a target neuron is a histogram whose entries count, for any positive or negative temporal delay $\Delta t$, the number of spikes that the target neuron emits with delay $\Delta t$ from any one spike of the reference neuron. If the target neuron tends to fire with delay $\Delta t$ before the reference ($\Delta t$ negative) or after it ($\Delta t$ positive), a peak in the CCH arises, centered at $\Delta t$. Other effects not related to correlated activity, such as firing rate variability and high regularity of the individual spike trains, may also cause peaks or oscillations in the CCH. Unbiased predictors of the CCH under the null hypothesis of spike train independence that account for these factors have been developed based on data surrogates (Louis et al. 2010c). For instance, a predictor accounting for both rate changes and spike regularity can be computed using a Monte Carlo approach as the average CCH obtained from surrogates of the original data generated by spike dithering. Confidence intervals can be obtained analogously.

Possible interactions among more than two spike trains are then obtained combining the information provided by the CCHs between all pairs. The proposed method works in three steps. Statistically significant pairwise correlations are determined on the basis of suitable predictors (for synchrony: at time lag $\Delta t=0$, or slightly larger to account for jitter). Second, cliques of all-to-all correlated pairs are collected, and all cliques above a preselected minimum size (e.g., all cliques of 3 or more neurons) are retained. Third, cliques sharing at least one neuron are merged into a single GIC.

Berger et al. (2007) applied this procedure to MPST data collected from cat V1 during visual stimulation with full field flash stimuli, and found four spatially clustered, distinct GICs comprising 3 to 21 neurons (Fig. 6d, each GIC shown in a different color). These GICs also formed clusters in cortical space and were speculated to reflect activity from underlying connectivity forming orientation columns as was shown by optical imaging (e.g., Hübener et al. 1997).

The method relies on the computation of the CCHs between all pairs of investigated neuronal activities and the evaluation of their statistical significance. The first amounts to $$\left( \begin{array}{l}{N}\\ {2}\\ \end{array}\right) $$ pairs for *N* neurons, a number that grows quadratically with *N*. Testing each CCH for significance using a Monte Carlo approach further requires the computation of up to hundreds of surrogate CCHs. The computational burden may become unaffordable without resorting on compute clusters. For this reason, Berger et al. (2010) worked out a pre-processing approach that excludes from the analysis individual neurons contributing weakly to synchronous events. The pre-processing step was used effectively on the same data and verified the original analysis, however at considerably reduced computational cost.

**Fig. 7:**
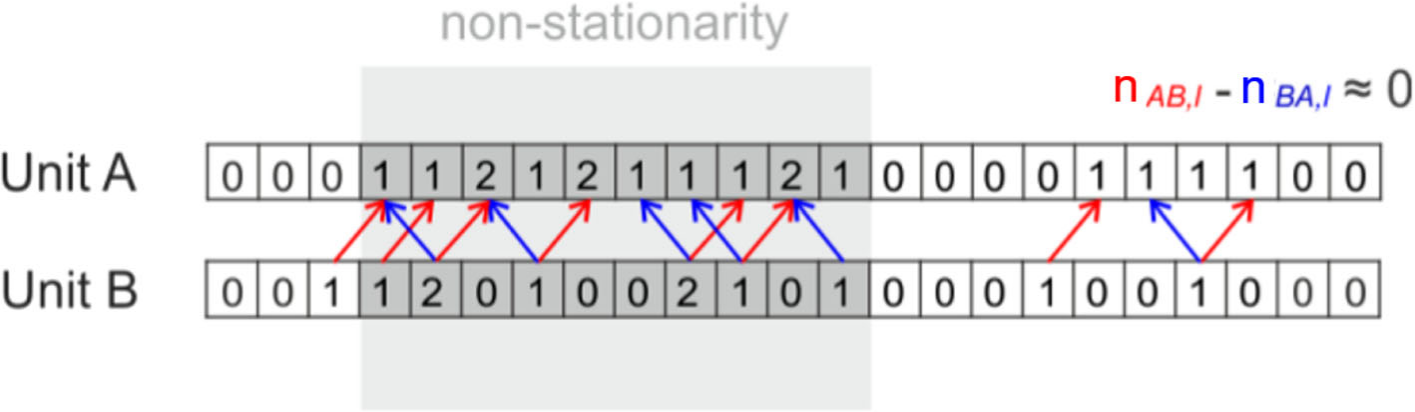
CAD pairwise test: sketch of the statistical pairwise test of the CAD method. The count $n_{AB,l}$ of spikes with a lag *l* is tested if it is significantly larger than the count at a reference lag. Here, the reference lag is chosen equal to $-\,l$, which correspond to the count $n_{AB,-l}=n_{BA,l}$. Under the null hypothesis of independent Poisson processes, the observable $n_{ABBA,l}:=n_{AB,l}-n_{BA,l}$ has average equal to 0 also in case of firing rate non-stationary firing rate (Reproduced with permission from Russo and Durstewitz 2017)

GICs formed by three or more neurons may be evidence for, but not necessarily imply, the presence of higher-than-pairwise correlation. The method does not test for genuine higher-order correlations (i.e., correlations that remain statistically significant when conditioning on correlations of lower order). The corresponding model of spiking activity is the pairwise correlated point process described in Sect. 2.2. On the other hand, higher-order correlations in the data may be, but not necessarily are, found as GICs.

#### Cell assembly detection (CAD)

Russo and Durstewitz (2017) recently introduced a different method to tackle the multiple testing problem arising in the search of repeated spike patterns in MPST data. The authors suggested an agglomerative algorithm (which we refer to here as cell assembly detection, or CAD) that is composed of two recursive steps: (a) a statistical test for pairwise correlations, and (b) a clustering procedure that agglomerates pairwise interactions into patterns of larger size. A very similar idea was introduced by Gerstein et al. (1978).

In step (a), spike trains are segmented in small time bins of width *w*. Then, for each pair (*A*, *B*) of spike trains, the algorithm counts the number $n_{AB,\bar{l}}$ of times that one spike of spike train *A* is followed by a spike of spike train *B* after $\bar{l}$ bins. The lag $\bar{l}$ is chosen to maximize the observed joint spike count $n_{AB,\bar{l}}$. Under the null hypothesis that the spike trains are realizations of independent Poisson processes, the method then derives the null distribution of the statistic

$$\begin{aligned} n_{ABBA,\bar{l}}=n_{AB,\bar{l}}-n_{AB,-l^{*}}, \end{aligned}$$

where $l^{*}$ is an arbitrary reference lag for which $n_{AB,\bar{l}}\ge n_{AB,l^{*}}$. Considering $n_{ABBA,\bar{l}}$ instead of $n_{AB,\bar{l}}$ is necessary to compensate for bias due to firing rate non-stationarity (see Fig. 7). If $n_{ABBA,\bar{l}}$ deviates significantly from 0, then the spike train pair *AB* is considered to be part of the same spike pattern. The advantage of this approach is that it avoids high computational cost by deriving all *p* values analytically. However, this strategy heavily relies on the assumption of Poissonianity which may not be a feature of the data and thus may lead to false positives (e.g., see Pipa et al. 2013). Also, $$\left( \begin{array}{l}{N}\\ {2}\\ \end{array}\right) $$ statistical tests are performed at this step in the presence of *N* spike trains, leading to a moderate multiple testing issue.

In step (b), larger spike patterns are obtained by recursively testing patterns previously formed with any other neuron, i.e., triplets are formed by testing each single significant pair *AB* with any other unit *C* using the same framework introduced for pairs. In order to make use of the null distribution derived for pairwise testing, all spikes of *A* with lag $\bar{l}_{AB}$ are considered to form a new artificial unit $(AB,\bar{l}_{AB})$, representing then the pattern occurrences. The test is then performed on the pair $((AB,\bar{l}_{AB})C,\bar{l}_{(AB)C})$. By proceeding iteratively with this agglomerative procedure, the algorithm extends from pairs to patterns of any size. Thus, this approach does not explicitly test for higher-order correlations, which leads to a lower statistical power than methods testing directly for higher-order correlations (see Sect. 5).

CAD can detect not only STPs, but also correlations of spike counts (e.g., firing rate modulation). To do so, the method allows the user to increase the bin size *w*, such that more than one spike is contained in a bin. For example in case that neuron *A* shows repeated increase in the firing rate, followed by an increase in neuron *B* after *l* bins (e.g., correlated non-stationary firing rates) appearing as spike count correlations in $n_{ABBA,\bar{l}}$. In particular, it is possible in the case of multiple spikes in the same bin to decompose each process in a sum of binary processes and to successively assess their significance using the same framework previously introduced. For additional details, we refer to the original publication. Thus, CAD is not limited to detect fine temporal spike pattern, but is also capable to detect correlations on a larger time scale.

**Fig. 8:**
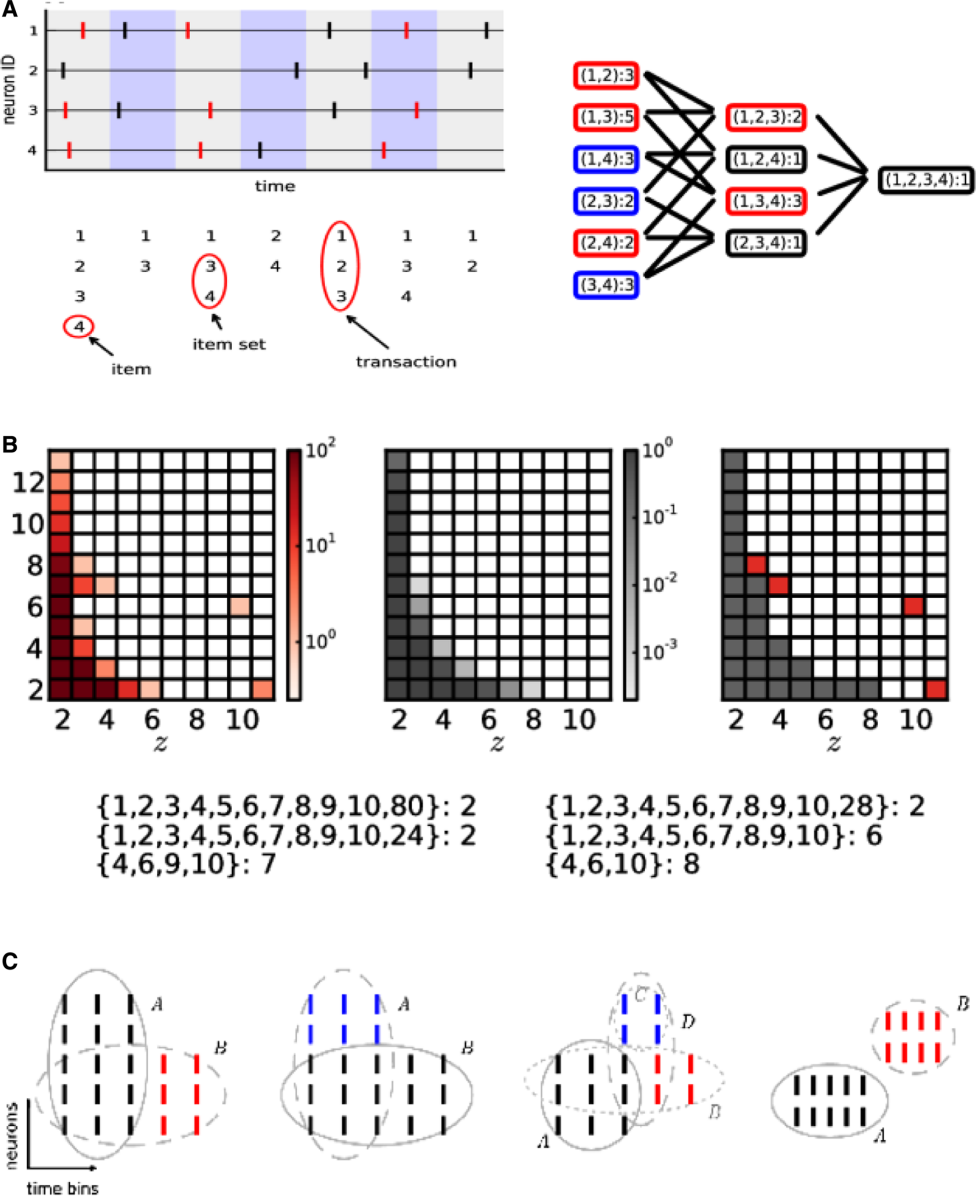
SPADE analysis. **a** Sketch of the discretization of the parallel spike data into binned spike trains. The set of neuron ids (“items”) spiking in each bin form a “transaction”. The subsets extracted from each transaction, or “item sets”, represent all observed synchronous spike patterns present in the data. The FIM data mining step organizes the item sets in a search tree and eventually returns all closed frequent item sets (right panel, circled in red), discarding the infrequent (black) and non closed (blue) ones. **b** Significance evaluation. Illustration of assessment of closed frequent patterns for statistical significance of simulated data consisting of a synchronous pattern of size $z=10$ occurring $c=6$ times and embedded in a population with 90 additional independent spike trains). From left to right: pattern spectrum of the number of patterns for each signature $(z,\,c)$ found in data; *p* value spectrum of each signature under the null hypothesis computed over statistically independent surrogates of the original data; significant (red) and non significant (gray) signatures in the original data (significance threshold: $\alpha =0.01$, corrected for multiple tests by false discovery rate correction). **c** Patterns found as statistically significant after PSF (lower lists in **b**) are tested for reciprocal conditional significance. Conditionally significant patterns are retained (here, the true pattern ${1,2,\ldots 10}$ occurring 6 times), the others are discarded as chance overlap of the significant ones with the background activity (Reproduced with permission from Torre et al. 2013)

#### Spike patterns detection and evaluation (SPADE)

Spike synchrony (see Sect. 2.3) or spatio-temporal spike patterns (Sect. 2.4) in MPST data can be effectively detected by the spike pattern detection and evaluation (SPADE) analysis method (see Torre et al. 2013; Quaglio et al. 2017, respectively). SPADE comprises three steps: (a) a data mining procedure to efficiently extract repeating synchronous spike patterns that are suitable candidates to be significant patterns, (b) statistical testing to assess the significance of the mined pattern candidates, and (c) assessment the conditional significance of each pattern retained after step (b), given any other found pattern overlapping with it; the last step is needed to reject patterns that are due to chance overlap of real patterns with background activity.

Step (a) is accomplished by Frequent Itemset Mining (FIM, Zaki and Ogihara 1998 or equivalently Formal Concept Analysis Ganter and Wille 1999; Pisková and Horváth 2013). Time is discretized into consecutive bins of duration *w*, and the sets of neurons emitting a spike in each bin are collected (see Fig. 8a). The activity of a synchronous cell assembly immersed in a larger population of recorded neurons (e.g., neurons 1, 3 and 4) typically appears as a set of spikes falling in the same time bin, together with additional spikes emitted by other neurons and falling in the same bin by chance. Revealing active synchronous cell assemblies thus requires to assess the statistical significance of all subsets of all transactions. However, for *N* neurons, the latter may be as many as $2^{N}$ different patterns, yielding severe computational and statistical issues. Of interest among these patterns are those which are *frequent*, i.e., occur at least a minimum number of times (in our case, 2 times), and which are *closed*, i.e., do not always occur as a subset of the same super-pattern. All patterns which are not frequent or not closed may be discarded under the rationale that they are either too sporadic, or trivially explained by larger patterns in the data. Figure 8a shows infrequent (black), frequent but not closed (blue) and frequent and closed (red) patterns extracted from the transactions in panel A. The latter are typically a small fraction of the total patterns. Therefore, testing them only for significance drastically reduces the computational burden and the multiple testing problem, without causing any information loss. FIM provides a class of efficient algorithms to collect closed frequent patterns in data of large size.

Similar approaches based on different, more heuristical data mining frameworks had been developed in previous work. See in particular Abeles and Gerstein (1988) and Gansel and Singer (2012) for two different algorithms to pre-filter patterns based on their neuronal composition. For an application of the former to MEG data, see Tal and Abeles (2016). These methods, however, do not guarantee that the filtered patterns are all closed (that is, all non-trivial) patterns in the data, thereby possibly leading to a loss of information. Also, neither of the two methodologies is accompanied by an approach to test for the statistical significance of the filtered patterns designed for MPST data.

Step (b) of SPADE, called pattern spectrum filtering (PSF; see Fig. 8b), assesses the statistical significance of each closed frequent pattern (typically thousands or more in MPST data) on the basis of the pattern size *z* (i.e., the number of neurons forming the pattern) and of the occurrence count *c* (i.e., the number of times the pattern occurs), irrespective of the specific neuronal composition of the pattern. The pair $(z,\,c)$ is called the pattern *signature*. Because the number of different pattern signatures is orders of magnitude smaller than the total number of different patterns, this pooling strategy avoids the multiple testing issue that would arise from testing each closed frequent pattern individually. PSF computes the *p* value of each observed signature based on surrogate data that preserve the marginal properties of the original spike trains such as the inter-spike intervals and the firing rate profiles (see Pipa et al. 2008; Louis et al. 2010c).

The presence of a real synchronous spike pattern in data tends to increase the occurrence count, and therefore the significance, of other patterns that result form a chance overlap of the pattern’s spikes with background activity. Step (c) of SPADE, called pattern set reduction (PSR) (see Fig. 8c), detects and removes these false positives by assessing the conditional significance of all patterns found after step (b) given any other overlapping one.

**Fig. 9:**
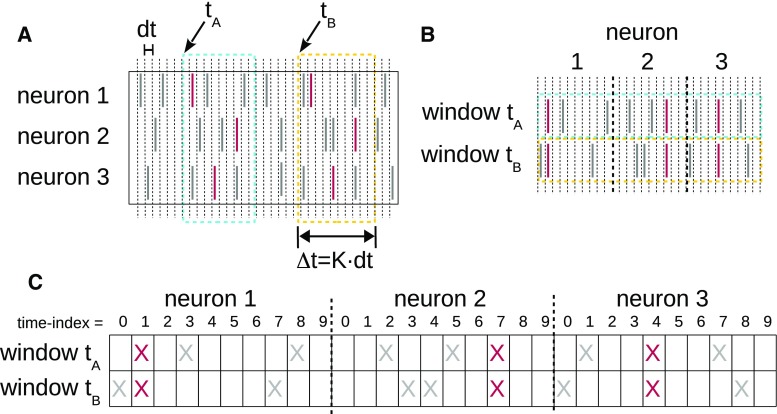
Detection of spatio-temporal spike patterns. **a** Construction of a transaction data base. Spike trains are binned, and a window of length *K* bins is slid in time in steps of 1 bin. For window positions which start with a spike, the spikes falling into the window are collected. These are transformed in time such that the spikes per neuron are concatenated to a vector such that a list of pairs (*i*, *j*) of spike id *i* and relative spike time *j*, $j=1,\ldots ,K$ are formed. **b** Transformed spiking activities from two window positions concatenated to parallel binary sequences enabling to search STPs by detection of synchronous entries as shown in **c** (Reproduced with permission from Quaglio et al. 2017)

Yegenoglu et al. (2016) and Quaglio et al. (2017) extended SPADE to detect arbitrary STPs (defined in Sect. 2.4). STPs spanning a maximum number of *K* bins (for synchrony: $K=1$) can be similarly defined as subsets of transactions constructed as follows. A window of *K* bins is slid through the data over time in steps of 1 bin (Fig. 9a). Each window position corresponds to a transaction whose elements (items) are pairs (*i*, *j*), one pair per spike in the window, *i* represents the id of the neuron that emitted the spike, while *j* represents the relative location of the spike inside the window ($j=1,\ldots ,K$) (Fig. 9b, c). Data formatted in transactions this way can be screened for closed frequent STPs by FIM (equivalently, FCA) algorithms. The evaluation of the statistical significance of closed frequent STPs requires the same steps as for synchronous patterns, namely PSF and PSR. Other approaches that filter patterns based on their stability (loosely speaking, the tendency of a pattern to reoccur identically) rather than on statistical significance were also investigated in Yegenoglu et al. (2016), but had a higher computational cost or yielded a lower performance.

**Fig. 10:**
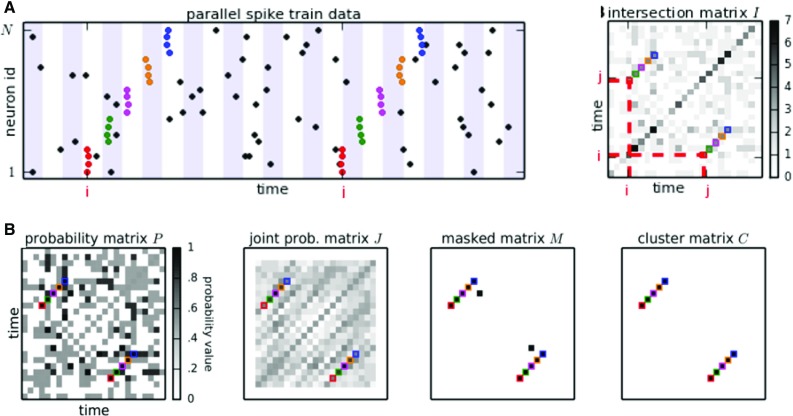
ASSET analysis. **a** Sketch of a raster plot of parallel spike trains of multiple neurons (vertical axis) over time (horizontal axis). Dots in each row correspond to the spike times of one neuron. Time is discretized into adjacent bins (marked by white and blue shaded backgrounds) to define synchronous events. Synchronous spikes forming an SSE repeating twice are indicated by colored dots (one color per event). On the right: Intersection matrix I. Each matrix entry $I_{i,j}$ (values encoded by gray levels) contains the degree of overlap of neurons active in time bins $b_{i}$ and $b_{j}$. **b** Significance evaluation of repeating SSEs. Left: The cumulative probability $P_{i,j}$ calculated for each entry $I_{i,j}$ analytically under the null hypothesis $H_{0}$ that the spike trains are independent and marginally Poisson. Second from left: The l largest neighbors of $I_{i,,j}$in a rectangular area extending along the 45$^{\circ }$ direction are isolated by means of a kernel and their joint cumulative probability is assigned to the joint probability matrix *J* at position $J_{i,j}$. Third from left: For a chosen significance threshold $\alpha _{1}$ for the probability of individual entries $P_{i,j}$ and a significance threshold $\alpha _{2}$ for the joint probability of the neighbors of entries $J_{i,j}$ each entry of *I* for which $P_{i,j}>\alpha _{1}$ and $J_{i,j}>\alpha _{2}$ is classified as statistically significant. Significant entries of *I* are retained in the binary masked matrix $M_{i,j}$, which takes value 1 at positions (*i*, *j*) where *I* is statistically significant and 0 elsewhere. B, right: 1-valued entries in M falling close-by are clustered together (or discarded as isolated chance events) by means of a DBSCAN algorithm, which thus isolates diagonal structures (Reproduced with permission from Torre et al. 2016a)

#### Analysis of sequences of synchronous events (ASSET)

Sequences of synchronous spike events (SSEs) constitute one type of coordinated spiking where synchrony propagates from one group of neurons to the next in a temporally precise manner. The synfire chain was proposed as one potential model for such kind of network processing. Torre et al. (2016a) introduced the Analysis of Sequences of Synchronous EvenTs (ASSET) to reveal this type of correlated activity in MPST data. The method builds on the work of Schrader et al. (2008), extending it by introducing statistical tests and thereby allowing for a fully automated analysis.

First, time is segmented into consecutive bins of length *w* (see Fig. 10a, left). Second, any two time bins are compared for the number of neurons that spike in these two bins, i.e., the intersection of the two sets. The results of all these comparisons form the intersection matrix *I* such that the comparison of bin *i* and *j* is entered in the matrix element $I_{i,j}$. Synchronous events composed of the same (or many overlapping) neurons lead to a larger value of $I_{i,,j}$ compared to independent data, i.e., chance overlap.

An SSE composed of (largely) the same neurons occurring two times in the data yields one diagonal structure of large entries in the intersection matrix *I*. Thus, a diagonal structure in the intersection matrix indicates the occurrence of a repeated SSE. ASSET detects and isolates diagonal structures in the intersection matrix by a statistical procedure. The method first transforms the intersection matrix *I* into a *probability matrix* *P* (Fig. 10b, left) defined such that $P_{ij}$ represents the probability for $I_{ij}$ to be at most the observed value, under the null hypothesis of spike train independence. $P_{ij}$ is obtained analytically or by Monte Carlo simulation. Values of $I_{ij}$ larger than expected correspond to values of $P_{ij}$ closer to 1. *P* is further transformed into a *joint probability matrix* *J* whose entries $J_{ij}$ represent the joint probability of overlaps all the intersections $I_{hk}$, where the bins *h*, *k* form a neighborhood of (*i*, *j*) (Fig. 10b, second from left). Diagonal structures in *I* due to a repeated SSEs lead to highly significant values both in *P* and in *J*. Individual, isolated repeated synchronous events yield a statistically significant entry in *P* but not in *J*. In light of these considerations, a *masked matrix* *M* is constructed, whose entries take binary values: $M_{ij}=1$ if both $P_{ij}$ and $J_{ij}$ are statistically significant, $M_{ij}=0$ otherwise (Fig. 10b, third from right). Finally, close-by one-valued entries in the masked matrix are clustered together in the cluster matrix *C* of diagonal structures. This step allows to identify the diagonal structures as individual entities, and to discard spurious isolated entries in *M* (Fig. 10b, right).

ASSET is robust to firing rate variability over time and across neurons, as well as to the presence of spike correlations different from SSEs (see Torre et al. 2016a). Furthermore, simulations of large balanced neuronal networks were used to demonstrate that the method is able to successfully discover SSEs resulting from repeated synfire chain activation.

## Method comparison 

In the previous sections, we gave an overview of nine methods for the analysis of temporally precise spike correlations in MPST data. We also illustrated the different ways these methods deal with the combinatorial and statistical challenges that characterize such an analysis. The various methods aim to reveal different types of correlated spiking activity. To this end, they rely on different statistics.

In the upcoming subsections, we give a comparative overlook of the applicability of these methods to data characterized by different correlation structures. In particular, we discuss the sensitivity of each method to correlations of a different type than the one it was designed to detect. A natural question here is whether a method designed to analysis a particular correlation structure may still provide partial information about different types of correlated spiking. If so, analyzing a data set with different methods may provide the researcher with a richer picture of the possibly present correlations, and even aid a correct interpretation of the results. In the following, we discuss how the introduced methods react to different correlation structures. Table 2 summarizes the results.

**Table 2:** Table of methods and stochastic models

Method	Pairwise correlation	Synchronous patterns	Population synchronization	STPs	SSEs
Complexity distribution	$(\checkmark )$	$(\checkmark )$	$\checkmark$		$(\checkmark )$
CuBIC	$(\checkmark )$	$(\checkmark )$	$\checkmark$		$(\checkmark )$
PUE	$(\checkmark )$	$(\checkmark )$	$\checkmark$		$(\checkmark )$
Max. entropy information	$\checkmark$		$\checkmark ^{*}$		
Max. entropy models	$\checkmark$	$\checkmark ^{*}$			
Cliques	$\checkmark$	$(\checkmark )$	$(\checkmark )$		$(\checkmark )$
SPADE	$(\checkmark )$	$\checkmark$	$(\checkmark )$	$\checkmark$	$\checkmark$
CAD	$(\checkmark )$	$\checkmark$	$(\checkmark )$	$\checkmark$	$\checkmark$
ASSET					$\checkmark$

The table summarizes the ability of each method to retrieve correlations represented by different models. $\checkmark$: the method is designed to detect that particular model and the output matches perfectly and describes completely the correlation structure of the data. $(\checkmark ):$ The method was designed for a different correlation model, but it is still possible to get partial information about the correlation structure of the data. $\checkmark ^{*}$: The method is in principle applicable, but in practice affected by computational and/or multiple testing issues when used on MPST data; the results may lead to misinterpret the correlation structure due to lack of information about it. For the remaining entries, the method does not provide sufficient information to reconstruct the correlation structure

### Population synchronization

In the case when synchronous spike events involve different neurons at each occurrence time, no particular spike pattern reoccurs. CuBIC and PUE find the minimum order of excess synchronous events to be assumed in the data. The test statistics are based on the complexity distribution, which does not include information about the neuronal composition of each synchronous event. PUE can additionally be performed in a time-resolved fashion, and therefore may discover time varying correlation orders. The CII approach quantifies the amount of information about the probability distribution of synchronous spike patterns that is delivered by correlations of a given order or lower, out of the total information delivered by all correlations. Thus, it may also be used to detect the maximum order of correlation in the data to account for a given percent (e.g., $99\%$) of such information. In practice though, CII is computationally intensive and typically cannot be used for MPST data to discriminate beyond second versus higher-order correlations.

The methods designed to detect specific groups of correlated neurons (MEMs, GIC, SPADE, CAD and ASSET), instead, are generally blind or weakly sensitive to population correlations. If the data are long enough and the population synchronization involves by chance the same spike patterns repeatedly, some of these methods may be able to classify such patterns as statistically significant. This, however, will provide only partial information about the true underlying correlation structure.

### Pairwise synchronization

The goal of the analysis of a data set containing pairwise synchronization consists in finding all the pairs of neurons involved in above-chance synchronous firing. In this scenario, CuBIC and PUE are expected to return the minimum order of correlation $\hat{\xi }$ necessary to explain the data, i.e., $\hat{\xi }=2$. This holds also true for the case of overlapping pairs of correlated neurons. However, if the total amount of synchronous spike pairs present in the data is not high enough, these methods may report spike train independence instead. However, since the identity of the neurons involved in synchronous firing is not resolved, the specific correlated pairs are not found. CII instead takes values very close to 1, thus highlighting the absence of higher-order correlations. In the presence of time varying spike train statistics, CuBIC is prone to report higher values of $\hat{\xi }$ because the method assumes stationary conditions. The PUE analysis and the CII, instead, can account for time varying rates (the former by a time-resolved analysis). For the CII approach, however, this comes at a significantly increased computational cost.

Among the considered methods for detection of cell assemblies, GIC and CAD directly evaluate the statistical significance of each pair of synchronous firing neurons. Testing only for pairwise interactions makes these methods particularly efficient (high statistical power, relatively low computational burden). GIC can also cope well with time varying firing rates, as suitable CCH predictors (surrogates) exist for this case (Louis et al. 2010b). However, it may fail in properly characterizing time varying pairwise correlations, since it relies on the cross-correlogram, which is a time average measure. CAD instead detect all the occurrences of the synchronous activation, allowing the reconstruction of the exact temporal evolution of the pairs synchronizations. Furthermore, the more relevant difference between GIC and CAD is that the first groups together pairs or neurons which are mutually correlated, while CAD, if the single occurrences of synchronization involves only pairs of neurons, does not group them, but it returns patterns formed by individual pairs. SPADE is designed to detect specific sets of correlated neurons, including pairwise synchronization. Its statistical power, however, is lower than that of GIC and CAD for pairwise synchronization. Indeed, SPADE first tests for pattern significance on the basis of the pattern size and occurrence count, irrespective of the neuronal composition. Thus, a specific pair has to exhibit a larger number of synchronous events to be detected as significant, compared to direct statistical tests.

Finally, ASSET cannot retrieve correlated pairs of neurons, because they do not produce repeated SSEs.

### Synchronous spike patterns

In the presence of synchronous spike patterns of size larger than 2, the optimal pattern detection would be a complete description of the correlated set of neurons: the neuron identities of the neurons in a synchronous event and their occurrence times.

In this scenario, methods to characterize population correlations (CuBIC, PUE, CII) generally tend to underestimate the correlation order in the data. The number of synchronous events of size $\xi$ or larger needed for these methods to report a minimum order $\xi$ of population correlation is much larger than the number of occurrences needed for a single pattern of size $\xi$ to become statistically significant. Unless several patterns of size $\xi$ exist, and their overall count is large enough, population methods will report correlations of lower order. This is particularly true for the CII index for $\xi =2$, which has been shown to take values very close to 1 (meaning that correlations of order 3 or higher contribute negligibly to the total information about the probability distribution of synchronous spike patterns) also when highly significant synchronous spike patterns of much larger size are present in the data (Torre 2016).

The GIC and CAD analysis may detect some of (but typically not all) the neurons forming a synchronous spike pattern of size larger than 2. The occurrences of the full pattern increase to some extent the peak in cross-correlations of the pairs contained in it, possibly leading to statistical significance for some of them. Only if all pairs become statistically significant, though, the two methods are guaranteed to further group them together and to reconstruct thereby the full pattern. This is typically not the case for patterns of larger size, since those typically exhibit lower occurrence counts in experimental data (see Torre et al. 2016b). An advantage of CAD, is the limited computational cost required to carry out the full analysis, due to the analytical formulation of the null distribution. Additionally, for CAD, the detected group forms a pattern which occurs multiple times with the same neural composition, while with GIC it is not possible to distinguish between actual spike pattern and a group of neurons that are mutually but independently correlated pairs.

MEM and SPADE are designed specifically to reliably detect reoccurring synchronous spike patterns, and therefore perform optimally in this scenario. MEM provides in addition a generative probabilistic model of the spiking activity, which allows for resampling. In addition, it allows one in principle to include correlations of any order among the neurons, as well as history effects that make the spike trains non-Poisson. On the down side, determining the model parameters becomes increasingly computationally demanding as more of such features are included. Also, testing for the statistical significance of each observed pattern runs into the multiple testing problem, effectively limiting the applicability of MEM to data with at most a few dozens of neurons. SPADE instead only indirectly conditions on existing correlations as it tests for the conditional significance of a pattern with a statistically significant signature given any other patterns overlapping with it. The method is also designed to drastically reduce the multiple testing issue. Importantly, it is very sensitive to synchronous events of large size, which need only few repetitions to reach the significance threshold. In contrast, low-order events need to occur more times to be identified as statistically significant (see Torre et al. 2013). A downside compared to MEM is that SPADE solely assesses pattern significance and does not provide a probabilistic model of the spiking activity.

ASSET, finally, does not detect isolated synchronous spike patterns (i.e., patterns not forming fixed, repeating sequences). The reason is that these events only produce isolated high-valued entries in the intersection matrix, but no diagonal structures.

### Spatio-temporal patterns

STPs are the generalization of synchronous patterns to the case when neurons fire in a fixed temporal order (yielding a synchronous spike pattern in the special case when the delays are 0). The general definition of STPs also includes SSEs as a special case. Methods designed to detect population synchronization (such as CuBIC, PUE, CII), as well as methods limited to the detection of spike synchrony (GIC, MEMs), are not sensitive to STPs (except, of course, for synchronous spike patterns). Methods like SPADE and CAD are able to identify STPs of the general type. Specifically, SPADE allows to correctly identify and statistically test for any repeating spike sequence with pre-assigned maximum time lag. No additional assumptions are made on the structure of the pattern. The same holds for CAD, where also the maximum time lag is fixed before the analysis and thus limited to the maximum allowed delay.

Finally, ASSET is only able to identify STPs of the SSE type, a special case which is discussed next.

### Sequences of synchronous spike events

An SSE consists of multiple synchronous events which occur at specific, fixed delays after one other. The presence of a reoccurring SSE (for instance due to the activation of an active synfire chain, see Sect. 2.5) thus increases the overall amount of synchronization observed in data. If the SSE comprises sufficiently many events or these events involve sufficiently many spikes, population correlation methods could therefore detect the presence of synchrony. If the size of all synchronous events in the SSE is the same, say $\xi$, CuBIC and PUE would ideally return synchronization order $\xi$ in the data. If instead the different synchronous events in the SSE have different size, they should return the maximum size. In both cases, however, both methods will typically return a lower correlation order. Furthermore, neither of the two methods identifies the neuronal composition of the events or their temporal structure. CII, instead, will report an information index $R_{2}$ very close to 1 if all events in the SSE comprise two spikes, and lower than if larger events are present. Computing indices $R_{\xi }$ for $\xi >2$ may help highlighting the existence of higher-order correlations, but it is computationally demanding. Besides, it would not provide a description of the complex correlation structure.

The GIC analysis could theoretically reconstruct the individual events forming an SSE. For this to be possible, the SSE has to occur sufficiently many times such that zero-delay pairwise correlations among all pairs of neurons involved in the same synchronous event become statistically significant. The method would then further group the overlapping pairs together, thus reconstructing each synchronous event separately. Besides that, even in this optimal scenario the synchronous events would be found in isolation, and further work would be needed to group them together into an SSE.

Since SSEs are a special case of STPs, they can be fully reconstructed with SPADE or CAD, if they occur sufficiently many times and if the total time span of one occurrence is shorter than the chosen analysis window. The number of occurrences needed for significance drops exponentially fast with the total number of involved neurons (see Torre et al. 2013). Thus, for SSEs involving sufficiently many neurons, even just a few repetitions are sufficient for detection by SPADE.

Finally, ASSET is specifically designed to detect SSEs occurring at least two times in the data. Unlike SPADE and CAD, the method accounts for their precise temporal structure (synchronous events and delays between them) to assess their significance. Specifically, ASSET computes the p value of the SSEs as the joint probability of having synchronous events of the observed size in sequence. SPADE instead computes the probability of having any STP of different composition comprising the same number of spikes. For this reason, the statistical power of ASSET for SSEs occurring two times is higher than that of SPADE. This allows ASSET to retrieve SSEs composed of fewer neurons than SPADE is able to discover. SPADE does, on the other hand, more easily detect SSEs occurring more than 2 times, because it collects evidence from all pattern occurrences. ASSET, instead, evaluates by default only the significance of pairs of SSE occurrences, unless intersection tensors of higher dimension are built (see Gerstein et al. 2012, for dimension 3), which is possible but computationally demanding.

## Discussion and conclusions

In this manuscript, we discussed methods which enable the analysis of massively parallel spike trains (the spiking activity of tens to hundred(s) of neurons recorded in parallel) for fine temporal correlations in the ms precision range. The common aim of such analyses is to identify spiking activity indicative of the presence of active cell assemblies (Hebb 1949), defined as groups of neurons that form building blocks for information processing in the cortex. Discovering and differentiating various types of temporally precise spike patterns in experimental spike data may be critical in understanding debated mechanisms of computations in the brain.

While no existing analysis method is able alone to distinguish among the different types of spike patterns discussed in the literature, combining the information delivered by different methods may provide a better strategy. Therefore, we suggest to apply multiple methods, in a particular sequence to approach unknown data. First, one would like to explore if there are at all indications for correlated activity. For doing that data can first be analyzed with computationally efficient methods, such as the complexity distribution (Grün et al. 2008) or other ’scanning’ methods (e.g., Berger et al. 2010). If the complexity distribution provides no indication for the presence of higher-order correlations, pairwise or low-order correlations or spatio-temporal patterns may still exist since the method is not sensitive for them. However, when correlations are found with the complexity distribution, or the maximum entropy methods, the sole interpretation is ’the data contain higher-order synchrony correlation’, or in case of the application of CuBIC ’the data contain HOC exceeding order X’. Only SPADE, CAD or ASSET allow to identify higher-order spike correlations including temporal delay between the spikes and they identify the neurons involved in. If such spatio-temporal patterns are found, their spatial occurrence on the recording array (e.g., Utah array) may be identified (e.g., Torre et al. 2016a). With additional knowledge on the detailed position of the array on the cortex potentially involved local areas and the propagation direction may be uncovered. If on the other hand recordings are performed directly from different areas, e.g., as in Zandvakili and Kohn (2015), ASSET may uncover the propagation of sequences of synchronous activity from area to area.

Depending on the protocol of the experiment and the behavioral design, data can be split into different trials or segments that allow different interpretation. If, for example, data are split and pooled according different behavioral conditions, the analysis of the two with the same method (e.g., SPADE) may result in the presence of different spike patterns, which may be interpreted as ’in behavior A a different assembly was activated than in behavior B’. Even more informative are time-resolved analysis approaches which can identify dynamically occurring spike patterns, as done in Riehle et al. (1997) and Kilavik et al. (2009) using the UE analysis. PUE, as a further development of the CuBIC analysis, enables also such a time-resolved analysis due to the low computational requirement. Other methods that have a higher computational load, such as SPADE or ASSET, can be applied in a pseudo time-resolved fashion by segmenting the full data into epochs of interest and pooling across trials. Different significant spike patterns may occur in different epochs or experimental condition, which may be interpreted as ’different cell assemblies are activated in different behavioral contexts’ (for an application of SPADE, see Torre et al. 2016b).

Experimental data typically exhibit various types of variability - non-stationary firing rates, rate inhomogeneity across neurons or trials, and inter-spike intervals being more or less irregular than a Poisson process are common observations. These features need to be included in the null hypothesis to avoid false positive findings (Grün et al. 2002, 2003; Pipa et al. 2013). However, an analytical description of the null hypothesis is for most of the cases mathematically not possible, or difficult in practice (for instance, parameters such as instantaneous firing rates cannot be well estimated from data; for a review see Grün 2009). Surrogate data, i.e., modifications of the original data obtained by destroying the aspect that is tested for, e.g., fine temporal correlations, provide a practical alternative solution (see Louis et al. 2010c; Grün 2009; Platkiewicz et al. 2017). For most of the methods discussed here, surrogates are used to derive the null distribution(s) in the presence of such non-stationarities. The downside is that this approach leads typically to a higher computational load.

The temporal resolution (binning) chosen for the analyses is a matter of choice, and may also be varied as a parameter for finding the relevant time scale. Furthermore, the discussed methods can be applied to data not consisting of parallel spike trains, such as continuous signals, as long as they can be reduced to point processes, and then to binary sequences by binning. This approach is common for calcium imaging data, which are typically reduced to events in time of the potential underlying spikes (Grewe et al. 2010). The time resolution is much lower than of electrophysiologically recorded spike data. However, the result is then a matter the interpretation. Another example are spike-like signals in MEG recordings (Abeles 2014). These were reduced in Tal and Abeles (2018) to point processes and can then be treated as binary processes and analyzed by the methods discussed in this review.

We compared the methods with respect to the correlation model they are designed for, and their abilities to detect other correlation structures. A quantitative comparison of the methods would likely provide more insights. However, we learned from previous pairwise comparisons of some of such methods (e.g., CAD and SPADE, Stella 2017, FIM and the accretion algorithm, Picado-Muiño et al. 2013) there are very few parameter configurations (e.g., temporal resolution, number of occurrences and size of the patterns or total length of the data) for which the performances are practically comparable. Moreover, the problem is not only about parameter configurations, but it is about the mathematical formulation, the different and not “hierarchical” definitions of correlated activity, which make a quantitative comparison difficult. A practical aspect for the difficulty of such comparisons is the fact that the various approaches are typically implemented in different software. A first step for an improvement of the situation would be a common software platform or even a common toolbox, as e.g., Elephant^1^.

However, one may not forget that the number of neurons recorded in parallel are still small compared to the number of neurons contained in the tissue under observation. For example, the number of neurons contained in a piece of cortex covered by, e.g., a 100 electrode Utah array (Blackrock Microsystems, Utah, USA) $(4 \times 4\,\text {mm}^{2})$ are about $10^{5}$–$10^{6}$. Thus sampling 100 or 200 neurons from the tissue is still sparse compared to the number of neurons therein. In addition, we still do not know how cell assemblies are spatially embedded. Thus, unfortunately, it is very likely that we still miss neurons from active assemblies. For improving this situation, a further increase in the number of neurons in parallel recorded should be aimed at and technically seems soon possible. This provides new opportunities to study large networks in even more details but will also require further extensions and developments of analysis methods.

## Electronic supplementary material

Below is the link to the electronic supplementary material.

Supplementary material 1 (pdf 52 KB)

## Footnotes

1
http://neuralensemble.org/elephant/

## References

[CR1] Abeles M (1982). Role of cortical neuron: integrator or coincidence detector?. Isr J Med Sci.

[CR2] Abeles M (1991). Corticonics: neural circuits of the cerebral cortex.

[CR3] Abeles M (2014). Revealing instances of coordination among multiple cortical areas. Biol Cybern.

[CR4] Abeles M, Gerstein GL (1988). Detecting spatiotemporal firing patterns among simultaneously recorded single neurons. J Neurophysiol.

[CR5] Aertsen A, Gerstein G, Habib M, Palm G (1989). Dynamics of neuronal firing correlation: modulation of "effective connectivity". J Neurophysiol.

[CR6] Anderson JA, Cooper L, Nass MM, Freiberger W, Grenander U (1995) Some properties of a neural model for memory. In: How we learn; how we remember: toward an understanding of brain and neural systems: selected papers of Leon N Cooper. World Scientific, pp 5–10

[CR7] Ashida G, Kretzberg J, Tollin DJ (2016). Roles for coincidence detection in coding amplitude-modulated sounds. PLoS Comput Biol.

[CR8] Bair W, Koch C (1996). Temporal precision of spike trains in extrastriate cortex of the behaving macaque monkey. Neural Comput.

[CR9] Bair W, Zohary E, Newsome W (2001). Correlated firing in Macaque visual area MT: time scales and relationship to behavior. J Neurosci.

[CR10] Berger D, Warren D, Normann R, Arieli A, Grün S (2007). Spatially organized spike correlation in cat visual cortex. Neurocomputing.

[CR11] Berger D, Borgelt C, Louis S, Morrison A, Grün S (2010) Efficient identification of assembly neurons within massively parallel spike trains. Comput Intell Neurosci 2010:1 10.1155/2010/439648 PMC2754663 19809521

[CR12] Bienenstock E (1995). A model of neocortex. Netw Compu Neural Syst.

[CR13] Borgelt C (2012). Frequent item set mining. Wiley interdisciplinary reviews (WIREs): data mining and knowledge discovery.

[CR14] Braitenberg V, Schüz A (1991). Anatomy of the cortex: statistics and geometry.

[CR15] Brown EN, Kass RE, Mitra PP (2004). Multiple neural spike train data analysis: state-of-the-art and future challenges. Nat Neurosci.

[CR16] Butts DA, Weng C, Jin J, Yeh C-I, Lesica NA, Alonso J-M, Stanley GB (2007). Temporal precision in the neural code and the timescales of natural vision. Nature.

[CR17] Buzsaki G (2004). Large-scale recording of neuronal ensembles. Nat Neurosci.

[CR18] Cunningham JP, Byron MY (2014). Dimensionality reduction for large-scale neural recordings. Nat Neurosci.

[CR19] Czanner G, Grün S, Iyengar S (2005). Theory of the snowflake plot and its relations to higher-order analysis methods. Neural Comput.

[CR20] Dayhoff JE, Gerstein GL (1983). Favored patterns in spike trains. I. Detection. J Neurophysiol.

[CR21] De Gruijl J, Hoogland T, De Zeeuw C (2014). Behavioral correlates of complex spike synchrony in cerebellar microzones. J Neurosci.

[CR22] Diesmann M, Gewaltig M-O, Aertsen A (1999). Stable propagation of synchronous spiking in cortical neural networks. Nature.

[CR23] Eggermont JJ (1990). The correlative brain, volume 16 of studies of brain function.

[CR24] Eggermont J (2015). Animal models of auditory temporal processing. Int J Psychophysiol.

[CR25] Ehm W, Staude B, Rotter S (2007). Decomposition of neuronal assembly activity via empirical de-poissonization. Electron J Stat.

[CR26] Elsayed GF, Cunningham JP (2017). Structure in neural population recordings: an expected byproduct of simpler phenomena?. Nat Neurosci.

[CR27] Fries P (2005). A mechanism for cognitive dynamics: neuronal communication through neuronal coherence. Trends Cogn Sci.

[CR28] Ganmor E, Segev R, Schneidman E (2015). A thesaurus for a neural population code. Elife.

[CR29] Gansel KS, Singer W (2012). Detecting multineuronal temporal patterns in parallel spike trains. Front Neuroinform.

[CR30] Ganter B, Wille R (1999). Formal concept analysis: mathematical foundations.

[CR31] Gerstein GL, Aertsen AMHJ (1985). Representation of cooperative firing activity among simultaneously recorded neurons. J Neurophysiol.

[CR32] Gerstein G, Clark W (1964). Simultaneous studies of firing patterns in several neurons. Science.

[CR33] Gerstein GL, Perkel DH, Subramanian KN (1978). Identification of functionally related neural assemblies. Brain Res.

[CR34] Gerstein GL, Bedenbaugh P, Aertsen A (1989). Neuronal assemblies. IEEE Trans Biomed Eng.

[CR35] Gerstein GL, Williams ER, Diesmann M, Grün S, Trengove C (2012). Detecting synfire chains in parallel spike data. J Neurosci Methods.

[CR36] Grewe BF, Langer D, Kasper H, Kampa BM, Helmchen F (2010). High-speed in vivo calcium imaging reveals neuronal network activity with near-millisecond precision. Nat Methods.

[CR37] Grün S (2009). Data-driven significance estimation of precise spike correlation. J Neurophysiol.

[CR38] Grün S, Diesmann M, Aertsen A (2002). Unitary Events in multiple single-neuron spiking activity. II. Non-stationary data. Neural Comput.

[CR39] Grün S, Riehle A, Diesmann M (2003). Effect of cross-trial nonstationarity on joint-spike events. Biol Cybern.

[CR40] Grün S, Abeles M, Diesmann M (2008) Impact of higher-order correlations on coincidence distributions of massively parallel data. In: Lecture notes in computer science, ’dynamic brain—from neural spikes to behaviors’, vol 5286, pp 96–114

[CR41] Harvey MA, Saal HP, III JFD, Bensmaia SJ (2013). Multiplexing stimulus information through rate and temporal codes in primate somatosensory cortex. PLoS Biol.

[CR42] Hebb DO (1949). The organization of behavior: a neuropsychological theory.

[CR43] Hoffman K, McNaughton B (2002). Coordinated reactivation of distributed memory traces in primate neocortex. Science.

[CR44] Hübener M, Shoham D, Grinvald A, Bonhoeffer T (1997). Spatial relationships among three columnar systems in cat area 17. J Neurosci.

[CR45] Izhikevich EM (2006). Polychronization: computation with spikes. Neural Comput.

[CR46] Jaynes ET (1957). Information theory and statistical mechanics. Phys Rev.

[CR47] Kass RE, Kelly RC, Loh W-L (2011). Assessment of synchrony in multiple neural spike trains using loglinear point process models. Ann Appl Stat.

[CR48] Kass RE, Eden UT, Brown EN (2014). Analysis of neural data.

[CR49] Kelly RC, Kass RE (2012). A framework for evaluating pairwise and multiway synchrony among stimulus-driven neurons. J Neural Comput.

[CR50] Kilavik BE, Roux S, Ponce-Alvarez A, Confais J, Gruen S, Riehle A (2009). Long-term modifications in motor cortical dynamics induced by intensive practice. J Neurosci.

[CR51] Kohn A, Smith MA (2005). Stimulus dependence of neuronal correlations in primary visual cortex of the Macaque. J Neurosci.

[CR52] König P, Engel AK, Singer W (1996). Integrator or coincidence detector? The role of the cortical neuron revisited. Trends Neurosci.

[CR53] Kuhn A, Rotter S, Aertsen A (2002). Correlated input spike trains and their effects on the response of the leaky integrate-and-fire neuron. Neurocomputing.

[CR54] Kuhn A, Aertsen A, Rotter S (2003). Higher-order statistics of input ensembles and the response of simple model neurons. Neural Comput.

[CR55] Kumar A, Rotter S, Aertsen A (2010). Spiking activity propagation in neuronal networks: reconciling different perspectives on neural coding. Nat Neurosci.

[CR56] Leen DA, Shea-Brown E (2012). A simple mechanism for higher-order correlations in integrate-and-fire neurons. BMC Neurosci.

[CR57] Levakova M, Tamborrino M, Ditlevsen S, Lansky P (2015). A review of the methods for neuronal response latency estimation. Biosystems.

[CR58] Lindsey BG, Morris KF, Shannon R, Gerstein GL (1997). Repeated patterns of distributed synchrony in neuronal assemblies. J Neurophysiol.

[CR59] Louis S, Borgelt C, Grün S (2010). Complexity distribution as a measure for assembly size and temporal precision. Neural Netw.

[CR60] Louis S, Borgelt C, Grün S (2010b) Generation and selection of surrogate methods for correlation analysis. In: Analysis of parallel spike trains. Springer, Boston, pp 359–382

[CR61] Louis S, Gerstein GL, Grün S, Diesmann M (2010). Surrogate spike train generation through dithering in operational time. Front Comput Neurosci.

[CR62] Murray JD, Bernacchia A, Freedman DJ, Romo R, Wallis JD, Cai X, Padoa-Schioppa C, Pasternak T, Seo H, Lee D, Wang X-J (2014). A hierarchy of intrinsic timescales across primate cortex. Nat Neurosci.

[CR63] Nicolelis MA (2001). Actions from thoughts. Nature.

[CR64] Ohiorhenuan IE, Mechler F, Purpura KP, Schmid AM, Hu Q, Victor JD (2010). Sparse coding and high-order correlations in fine-scale cortical networks. Nature.

[CR65] Palm G (1981). Evidence, information and surprise. Biol Cybern.

[CR66] Perkel DH, Gerstein GL, Moore GP (1967). Neuronal spike trains and stochastic point processes II. Simultaneous spike trains. Biophys J.

[CR67] Perkel DH, Gerstein GL, Smith MS, Tatton WG (1975). Nerve-impulse patterns: a quantitative display technique for three neurons. Brain Res.

[CR68] Picado-Muiño D, Borgelt C, Berger D, Gerstein GL, Grün S (2013). Finding neural assemblies with frequent item set mining. Front Neuroinform.

[CR69] Pipa G, Munk MHJ (2011). Higher order spike synchrony in prefrontal cortex during visual memory. Front Comput Neurosci.

[CR70] Pipa G, Wheeler DW, Singer W, Nikolic D (2008). Neuroxidence: reliable and efficient analysis of an excess or deficiency of joint-spike events. J Neurosci Methods.

[CR71] Pipa G, Grün S, van Vreeswijk C (2013). Impact of spike train autostructure on probability distribution of joint spike events. Neural Comput.

[CR72] Pisková L, Horváth T (2013) Comparing performance of formal concept analysis and closed frequent itemset mining algorithms on real data. In: CLA, pp 299–304

[CR73] Platkiewicz J, Stark E, Amarasingham A (2017). Spike-centered jitter can mistake temporal structure. Neural Comput.

[CR74] Price NS, Born RT (2010). Timescales of sensory- and decision-related activity in MT and MST. J Neureosci.

[CR75] Prut Y, Fetz EE (1999). Primate spinal interneurons show pre-movement instructed delay activity. Nature.

[CR76] Prut Y, Vaadia E, Bergman H, Haalman I, Hamutal S, Abeles M (1998). Spatiotemporal structure of cortical activity: properties and behavioral relevance. J Neurophysiol.

[CR77] Quaglio P, Yegenoglu A, Torre E, Endres DM, Grün S (2017). Detection and evaluation of spatio-temporal spike patterns in massively parallel spike train data with spade. Front Comput Neurosci.

[CR78] Reimer I, Staude B, Ehm W, Rotter S (2012). Modeling and analyzing higher-order correlations in non-poissonian spike trains. J Neurosci Methods.

[CR79] Riehle A, Grün S, Diesmann M, Aertsen A (1997). Spike synchronization and rate modulation differentially involved in motor cortical function. Science.

[CR80] Riehle A, Kornblum S, Requin J (1997). Neuronal correlates of sensorimotor association in stimulus-response compatibility. J Exp Psychol Hum Percept Perform.

[CR81] Riehle A, Wirtssohn S, Grün S, Brochier T (2013). Mapping the spatio-temporal structure of motor cortical lfp and spiking activities during reach-to-grasp movements. Front Neural Circuits.

[CR82] Rostami V (2017) Statistical analysis tools for assessing the functional relevance of higher-order correlations in massively parallel spike trains. Ph.D. thesis, RWTH Aachen University

[CR83] Rostami V, Mana PP, Grün S, Helias M (2017). Bistability, non-ergodicity, and inhibition in pairwise maximum-entropy models. PLoS Comput Biol.

[CR84] Roudi Y, Nirenberg S, Latham PE (2009). Pairwise maximum entropy models for studying large biological systems: when they can work and when they can’t. PLoS Comput Biol.

[CR85] Russo E, Durstewitz D (2017). Cell assemblies at multiple time scales with arbitrary lag constellations. eLife.

[CR86] Schneidman E, Still S, II MJB, Bialek W (2003). Network information and connected correlations. Phys Rev Lett.

[CR87] Schneidman E, Berry MJ, Segev R, Bialek W (2006). Weak pairwise correlations imply strongly correlated network states in a neural population. Nature.

[CR88] Schrader S, Grün S, Diesmann M, Gerstein G (2008). Detecting synfire chain activity using massively parallel spike train recording. J Neurophysiol.

[CR89] Schultze-Kraft M, Diesmann M, Gruen S, Helias M (2013). Noise suppression and surplus synchrony by coincidence detection. PLoS Comput Biol.

[CR90] Schwarz DA, Lebedev MA, Hanson TL, Dimitrov DF, Lehew G, Meloy J, Rajangam S, Subramanian V, Ifft PJ, Li Z, Ramakrishnan A, Tate A, Zhuang KZ, Nicolelis MAL (2014). Chronic, wireless recordings of large-scale brain activity in freely moving rhesus monkeys. Nat Methods.

[CR91] Seki S, Eggermont JJ (2003). Changes in spontaneous firing rate and neural synchrony in cat primary auditory cortex after localized tone-induced hearing loss. Hear Res.

[CR92] Shimazaki H, Amari S-I, Brown EN, Grün S (2012). State-space analysis of time-varying higher-order spike correlation for multiple neural spike train data. PLoS Comput Biol.

[CR93] Shlens J, Field GD, Gauthier JL, Grivich MI, Petrusca D, Sher A, Litke AM, Chichilnisky E (2006). The structure of multi-neuron firing patterns in primate retina. J Neurosci.

[CR94] Staude B, Rotter S, Grün S (2010). Cubic: cumulant based inference of higher-order correlations in massively parallel spike trains. J Comput Neurosci.

[CR95] Staude B, Grün S, Rotter S (2010). Higher-order correlations in non-stationary parallel spike trains: statistical modeling and inference. Front Comput Neurosci.

[CR96] Stella A (2017) Comparison of statistical methods for spatio-temporal patterns detection in multivariate point processes: an application to neuroscience. Master’s thesis, University of Turin

[CR97] Strangman G (1997). Detecting synchronous cell assemblies with limited data and overlapping assemblies. Neural Comput.

[CR98] Swadlow HA (1994). Efferent neurons and suspected interneurons in motor cortex of the awake rabbit: axonal properties, sensory receptive fields, and subthreshold synaptic inputs. J Neurophysiol.

[CR99] Tal I, Abeles M (2016). Temporal accuracy of human cortico–cortical interactions. J Neurophysiol.

[CR100] Tal I, Abeles M (2018). Imaging the spatiotemporal dynamics of cognitive processes at high temporal resolution. Neural Comput.

[CR101] Tang A, Jackson D, Hobbs J, Chen W, Smith JL, Patel H, Pietro A, Petrusca D, Grivich MI, Sher A, Hottowy P, Dabrowski W, Litke AM, Beggs JM (2008). A maximum entropy model applied to spatial and temporal correlations from cortical networks in vitro. J Neurosci.

[CR102] Tetzlaff T, Rotter S, Aertsen A, Diesmann M (2007) Time scale dependence of neuronal correlations. In: Göttingen Meeting of the German Neuroscience Society, Göttingen

[CR103] Tetzlaff C, Kolodziejski C, Markelic I, Wörgötter F (2012). Time scales of memory, learning, and plasticity. Biol Cybern.

[CR104] Tetzlaff C, Dasgupta S, Kulvicius T, Wörgötter F (2015). The use of hebbian cell assemblies for nonlinear computation. Sci Rep.

[CR105] Tkacik G, Schneidman E, II MJB, Bialek W (2006) Ising models for networks of real neurons. arXiv:q-bio/0611072

[CR106] Torre E (2016) Statistical analysis of synchrony and synchrony propagation in massively parallel spike trains. Ph.D. thesis, RWTH AAchen

[CR107] Torre E, Picado-Muiño D, Denker M, Borgelt C, Grün S (2013). Statistical evaluation of synchronous spike patterns extracted by frequent item set mining. Front Comput Neurosci.

[CR108] Torre E, Canova C, Denker M, Gerstein G, Helias M, Grün S (2016). ASSET: analysis of sequences of synchronous events in massively parallel spike trains. PLoS Comput Biol.

[CR109] Torre E, Quaglio P, Denker M, Brochier T, Riehle A, Grün S (2016). Synchronous spike patterns in macaque motor cortex during an instructed-delay reach-to-grasp task. J Neurosci.

[CR110] Vaadia E, Haalman I, Abeles M, Bergman H, Prut Y, Slovin H, Aertsen A (1995). Dynamics of neuronal interactions in monkey cortex in relation to behavioural events. Nature.

[CR111] Yegenoglu A, Quaglio P, Torre E, Grün S, Endres D (2016) Exploring the usefulness of formal concept analysis for robust detection of spatio-temporal spike patterns in massively parallel spike trains. In: Graph-based representation and reasoning. Springer, pp 3–16

[CR112] Zaki MJ, Ogihara M (1998) Theoretical foundations of association rules. In: 3rd ACM SIGMOD workshop on research issues in data mining and knowledge discovery

[CR113] Zandvakili A, Kohn A (2015). Coordinated neuronal activity enhances corticocortical communication. Neuron.

