# Supplementary material for: Methods for identification of spike patterns in massively parallel spike trains
Source: Biol Cybern. 2018 Apr 12;112(1):57–80. doi: 10.1007/s00422-018-0755-0 (PMC5908877; doi:10.1007/s00422-018-0755-0)
Supplement: Supplementary file 1 — Supplementary material 1 (pdf 52 KB) [file 422_2018_755_MOESM1_ESM.pdf]

6 Supplementary Material

6.1 Point Processes models for Correlated Spike Trains

The correlated parallel spike trains models described in section §2 can be formalized as Marked Point Processes (MPP). We can consider an MPP $M(t)$ where the k-th event is marked with a random variable P_k, which corresponds to one subset of the set $\{1, \ldots, n\}$, where n is to the total number of neurons. P_k consists of the set of indexes of the neurons that fires simultaneously at that point in time. The firing times of the i-th neuron can be described by the counting process $X_i(t) = N_i(t) + M_i(t)$ where $N_i(t)$ counts the individual spikes of neuron i occurring with background rate λ_i and $M_i(t) = M(t) \cdot \mathbb{1}_{\{i \in P_k\}}$ gives the spikes due to synchronous activity. If $M(t)$ and all $N_i(t)$ are independent Poisson processes then the population process $Z(t) = \sum_{i=1}^{n} X_i(t)$ (sum of the spikes of all the neurons) is a compound Poisson process $\mathrm{CPP}(N(t), A_k)$, where $N(t)$ counts the total number of events until time t and A_k indicates the number of synchronous spikes in the k-th event, that is, either one if the event is a single neuron firing and otherwise the size of the corresponding set S_k . The complexity of the process is the size of the largest possible set.

Using this framework, it is possible to derive a formal model for each of the correlation structures introduced in section §2.

The population synchronization corresponds exactly to the model just introduced, where P_k can correspond to any possible subset of the set $\{1, \ldots, n\}$. The pairwise synchronization can be modeled by considering P_k assuming all and only values in the set of possible pairs (i, j) with $i, j \in \{1, \ldots, n\}$. For the synchronous pattern P_k is fixed for every event and it represents the set of the indexes of the neurons involved in the pattern. For the spatio-temporal pattern we need to additionally define a set of delays $\{\delta_0, \ldots, \delta_\xi\}$ that represents the lags between the first and each of the other spikes forming the pattern, such that $M_i(t) = (M(t) + \delta_i) \cdot \mathbb{1}_{\{i \in A_k\}}$. Similarly for the SSEs we can define the neuronal sets forming the successive layers of synchronous spikes $\{L_{1,} \ldots, L_l\}$ and their respective delays $\{\delta_0, \ldots, \delta_l\}$, such that $P_k = \{L_1 \cup \ldots \cup L_l\}$in order to define the sequences by $M_i(t) = (M(t) + \delta_j) \cdot \mathbb{1}_{\{i \in L_j\}}$.
